# The Site of Azido Substitution in a Pyrimidine Nucleobase Dictates the Type of Nitrogen-Centered Radical Formed after Dissociative Electron Attachment

**DOI:** 10.1021/acs.jpcb.5c02751

**Published:** 2025-08-04

**Authors:** Daniel Adjei, Maria de Cabrera, Yahaira Reyes, Alexandru Barbolovici, Moaadh Alahmadi, Samuel Ward, Marie-Claude Menet, Philippe Mejanelle, Anil Kumar, Michael D. Sevilla, Stanislaw F. Wnuk, Mehran Mostafavi, Amitava Adhikary

**Affiliations:** † Department of Chemistry and Biochemistry, 5450 Florida International University, Miami, Florida 33199, United States; ‡ 129954 Institut de Chimie Physique, UMR 8000 CNRS, Bât. 349,Université Paris-Saclay; 91405 Orsay, Cedex, France; § Department of Chemistry, 146 Library Drive, 6918 Oakland University, Rochester, Michigan, 48309, United States; ∥ IUT d’Orsay, 13 Avenue des Sciences, 91190, Gif-sur-Yvette, France; ⊥ Free Radical and Radiation Biology Program, 4216 Medical Education Research Facility, Department of Radiation Oncology, Carver College of Medicine, 4083 University of Iowa, 375 Newton Road, Iowa City, Iowa 52242, United States

## Abstract

Azido­(N_3_)-nucleosides are known for their antiviral and anticancer properties. Recently, N_3_-nucleosides have attracted attention for augmenting radiation damage in tumor cells via dissociative electron attachment (DEA) reactions mediated by the simplest and the most potent reducing species (the electron). To investigate the effect of N_3_ group substitution at specific sites of the pyrimidine base-ring (C4, C5, and C6) on the DEA reaction, we employed both commercially available and in-house-synthesized N_3_-nucleosides. We conducted a comprehensive study combining electron paramagnetic resonance (EPR) spectroscopy at a low temperature, picosecond pulse radiolysis in an aqueous solution under ambient conditions, and DFT calculations. For N_3_ substitution at C4, EPR studies and DFT calculations established a stable azide anion radical (R–N_3_^•–^) formation, after the addition of radiation-produced electrons. For N_3_ substitution at C5, the DEA leads to a π-type aminyl radical (R–NH^•^) formation. For N_3_ substitution at C6, a conjugated iminyl σ-radical (R=N^•^) is formed via DEA. R=N^•^ is in equilibrium with R–NH^•^. Thus, this work reports a significant finding: the stabilization and reactivity of each type of nitrogen-centered radical (RN_3_^•–^, RNH^•^, R=N^•^) are determined by the position of N_3_ substitution on the pyrimidine base-ring.

## Graphical abstract

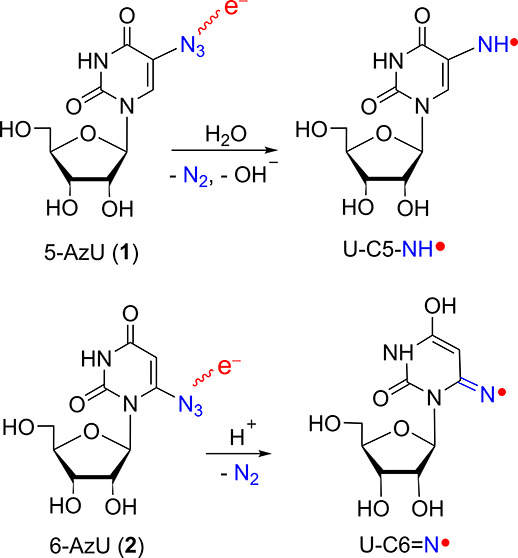

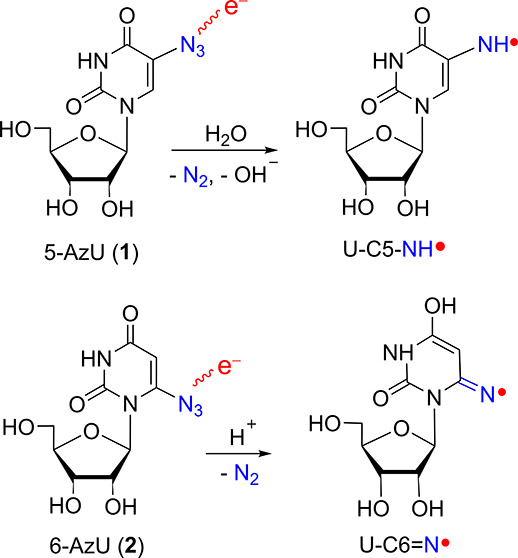

## 1 Introduction

The first organic azide, phenyl azide, was discovered by Grieβ around 160 years ago. Since then, many organic azides have found widespread applications.^1^ For over six decades, azido (N_3_)-modified nucleosides have demonstrated versatile applications in biochemistry, ranging from fundamental research to therapeutic development.^2,3^ Key applications include: (i) click chemistry, (ii) bioconjugation and ligation, (iii) aminonucleoside synthesis, (iv) metabolic labeling of DNA and RNA and for live cell imaging, and (v) enzyme inhibition (e.g., inhibition of ribonucleotide reductase (RNR)) that leads to their antiviral and anticancer actions.^1–7^ Owing to systematic investigations on N_3_-compounds,^3,8–10^ the application of N_3_-nucleosides as radiosensitizers emerged and demonstrated significant promise.^11^

All ionizing radiation processes lead to copious amount of free electrons after a cascade of ionization events.^8^ Electrons, being the simplest and strongest reducing species, can augment DNA damage through electron-induced reactions, leading to radiosensitization.^8–18^ Electron paramagnetic resonance (EPR) has been extensively employed to investigate these electron-induced reactions.^8^ EPR studies of irradiated DNA–protein complexes, nucleohistones, and chromatin have established that facile electron transfer occurs significantly from protein radicals to the electron-affinic pyrimidine bases within DNA, viz., thymine (T) and cytosine (C).^19–23^

This electron transfer pathway provides an effective strategy of radiosensitization using electron-affinic analogs of these bases. C5 Substitution in T is often chosen as these T-analogs can be readily incorporated in DNA.^8–14,17,24–34^ For example, substitution of CH_3_ with a strong electrophile (e.g., Br) is an effective strategy in radiosensitizer design.^8–14,17,24–28^ After incorporation in DNA, these modified analogs (e.g., 5-bromo-2′-deoxyuridine (5BrdU)) undergo dissociative electron attachment (DEA) to form highly reactive nucleobase radicals that cause subsequent reactions (e.g., H-atom abstraction, addition to –C=C−). These processes augment DNA damage products, which eventually lead to radiation-induced cell death.^3,8–14,17,24–35^

5BrdU cannot be applied in a clinical setting due to severe normal tissue toxicity associated with its delivery.^9,12–18,24–28,36–40^ So, current research focuses on synthesizing new C5-modified Thd derivatives that are effective in the DEA region of 0–3 eV and induce significant extent of single strand breaks in the nuclear DNA of proliferating cells along with considerably less C5-substituent loss via thymidylate synthetase. Thus, it is important that the C5 substitution in Thd still remains intact after cellular incorporation.^3,8–11,24–26,37–40^ However, previous work showed that 5-AzidoPyr derivatives maintained cell viability even at 100 μm, thereby avoiding the drawback of 5BrdU.^11^ In addition, exposure of HeLa cells in the presence of 0.1 mM aqueous solution of 3′-azidothymidine (3′-AZT or zidovudine) showed a direct correlation between cell death as well as micronuclei-induction under increasing doses of γ-irradiation.^41^ 3′-AZT has been shown to induce significant radiosensitization in irradiated other cancer cell lines, including human colon cancer cells,^42^ in irradiated human larynx squamous carcinoma cells,^43^ in irradiated human malignant glioma cells,^44^ and EBV (Epstein–Barr virus)-transformed lymphoblastoid cells in vitro.^45^ However, radiosensitization by 3′-AZT against these tumor cell lines does not exclude the possibility that (a) 3′-AZT is metabolized in the liver or (b) it may have toxicity against normal tissues.

Employing several C5-azidomethyl and C5-azidovinyl-Pyr nucleosides, a previous work showed that C5-azidomethyl-2′-deoxyuridine (5-AZmdU) induced significant radiosensitization in EMT-6 breast cancer cells.^11^ Enzymatic assays confirmed 5-AZmdU incorporation in dsDNA (e.g., in Klenow fragment polymerization).^11^ Similarly, incorporating an azido (N_3_) group into sesquiterpene lactones (e.g., parthenolide and dehydroleucodine) led to a significant suppression of proliferation rate and clonogenic survival of irradiated MCF-7 cells.^46^

Employing in-house-synthesized isotopically labeled (^15^N, deuterium) azidoPyr nucleosides and azidosugars, EPR studies at low temperatures, DFT calculations, as well as pulse radiolysis (ELYSE) in aqueous solutions provided unequivocal evidence of NCR (nitrogen-centered (N-centered) radicals) formation through DEA via radiation-produced electrons.^3,8,10,11,46–49^ Previous work demonstrates that electron addition to azido-compounds first produces the transient negative ion (TNI), i.e., the azide anion radical, R–N_3_^•–^. R–N_3_^•–^, undergoes DEA with N_2_ loss and forms the highly basic nitrene anion radical, RN^•–^.^3,8,10,11,46–49^ Even at high pH (pH ca. 12), RN^•–^ rapidly protonates from the surrounding water to form RNH^•^.^3,8,10,11,46–49^ The π-type RNH^•^ in which the radical site N-atom is attached to a saturated primary carbon (i.e., methylene (CH_2_) site) undergoes facile conversion to σ–R=N^•^ via an α-azidoalkyl radical intermediate. This is observed in 5-azidomethyl-2′-deoxyuridine (5-AmdU) or its 5′-azidomethyl isomer.^3,8,10,11,49^ Moreover, RNH^•^ undergoes facile tautomerization to form R=N^•^ when the radical site N-atom is attached to an unsaturated C-atom, e.g., in 5-(1-azidovinyl)-2′-deoxyuridine.^11^ A comprehensive review of NCR formation and reactivity from azidonucleosides and related compounds has recently been published.^3^

Recent work has shown nitrogen (N_2_) loss on DEA to form an NCR as a general mechanism but with the single exception of 6-azidomethyluridine (6-AmU).^10^ In case of 6-AmU, facile formation of a neutral C-centered allylic radical (U-6-CH_2_^•^) accompanied by an azide anion (N_3_^–^) was observed through DEA from the TNI (U-6-CH_2_–N_3_^•–^).^10^ The TNI could not be detected by EPR (77 K) or ELYSE (room temperature).^10^

In this current work, we investigated the reactions of radiation-produced electrons with commercially available 5-azidouridine (**1**), in-house-synthesized 6-azidouridine (**2**), a 4-azido-2′-deoxycytidine analog (**3**), and a tetrazole analog of 2′-deoxycytidine (**4**). In **4**, the N_3_ group attached to ring C4 is covalently linked to the ring N3 atom (Scheme 1). Following several previous studies,^10,11^ we are interested in the nucleobase-NCR only. We employed two complementary experimental techniques: EPR (low temperature) and pulse radiolysis (ELYSE, at room temperature). We have also used DFT calculations to predict EPR parameters (hyperfine coupling constant (HFCC) values) for comparison with the corresponding experimentally determined ones. We performed ion chromatography to check the possibility of N_3_^–^ release on γ-irradiated aqueous solutions of **1**.

**Scheme 1:**
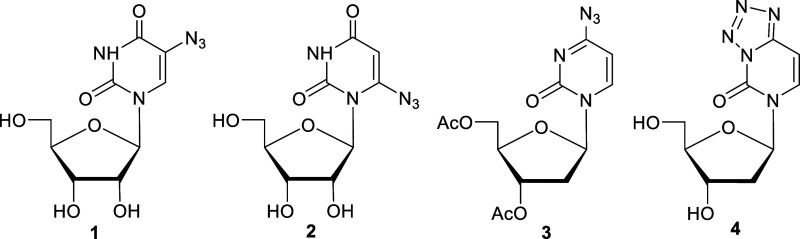
Structures of Azidonucleosides (**1** (5-Azidouridine), **2** (6-Azidouridine), **3** (4-Azido-2′-deoxycytidine Analog), and **4** (Tetrazole Analog of 2′-Deoxycytidine)) Investigated in This Work

For N_3_ substitution on Pyr base-ring systems (Scheme 1), the formation of various NCRs is observed: a stable azide anion radical, Pyr–N_3_^•–^ (from **3** and **4**), a π-type aminyl radical, Pyr–NH^•^ (from **1**), and a conjugated iminyl σ-radical, Pyr=N^•^, in equilibrium with Pyr–NH^•^ (from **2**). These results differ from previously published findings involving azidomethyl groups (−CH_2_N_3_) directly attached to C5 (5-AmdU) and C6 (6-AmU) of the Pyr base moiety^3,10,11^ as well as to the C5′ position of the 2′-deoxypentofuranose sugar moieties in Pyr nucleosides.^3,11,47–49^ Consequently, this comparison highlights the influence of the –CH_2_ group attached to the N_3_ moiety on the DEA, affecting the DEA process from the TNI, and consequently, the type and reactivity of the resulting NCRs. This work, therefore, reports a significant finding that the position of direct N_3_ moiety substitution on the Pyr base-ring stabilizes the specific type of NCRs (RN_3_^•–^, RNH^•^, R=N^•^). Also, the anion chromatographic studies established that N_3_® release does not happen in **1**, thereby validating the DEA pathway proposed in previous work.^3,11,47–49^

## 2 Materials and Methods

5-Azidouridine (**1**) was obtained from Biosynth, UK (formerly known as Carbosynth). Following previous work,^3,8,10,11,46–49^ (a) synthesis and spectroscopic characterization of **2**, **3**, **4**; (b) EPR studies, (c) theoretical calculations, and (d) ion chromatography are presented in the Supporting Information section.

## 3 Results and Discussion

### 3.1 Synthesis

6-Azidouridine (**2**) was prepared following the reported procedures.^50,51^ Synthesis of 3′,5′-di-*O*-acetyl-4-azido-2′-deoxycytidine (**3**, 1-(3,5-di-*O*-acetyl-2-deoxy-β-d-ribofuranosyl)-4-azidopyrimidin-2­(1*H*)-one) and 4-azido-2′-deoxycytidine tetrazolo tautomer (**4**, 6-(2-deoxy-β-d-ribofuranosyl)­tetrazolo­[l,5-c]­pyrimidin-5­(6*H*)-one) is described in the Supporting Information section.

### 3.2 EPR Studies

#### 3.2.1 EPR Spectral Studies of One-Electron Addition to 5-Azidouridine (**1**, 5-AzU) Forming a π-Type Aminyl Radical, RNH^•^

First derivative EPR spectra of the radicals formed from matched γ-irradiated (absorbed dose = 600 Gy at 77 K) homogeneous glassy (or supercooled homogeneous) 7.5 M LiCl/D_2_O (green, Figure 1A) and 7.5 M LiCl/H_2_O (red, Figure 1B) solutions of commercially available compound **1** ([**1**] = 2 to 3 mg/mL) are presented in Figure 1. EPR spectra were recorded at 77 K. The line components due to Cl_2_^•–^ were subsequently subtracted from these spectra. We note that Cl_2_^•–^ is produced by scavenging of radiation-produced holes in the matrix (7.5 M LiCl).^3,8,10,11,46–49^ Each of these two spectra represents a triplet due to an axially symmetric anisotropic aminyl ^14^N (spin = 1) with a hyperfine coupling constant, HFCC, (***A***_*zz*_ (***A***_∥_) = 20 G, ***A***_⊥_ = ***A***_*xx*_ = ***A***_*yy*_ = 0 G), along with *g*_∥_ = 2.0020 and *g*_⊥_ = 2.0043. A comparison of the total widths of these two spectra shown in Figure 1A (green) and 1B (red) demonstrates the presence of an additional ca. 17 G hyperfine coupling in the radical formed in the H_2_O glasses (red, Figure 1B). This coupling is absent in the D_2_O glasses (green, Figure 1B). Thus, this result establishes that the additional 17 G of hyperfine coupling is due to the ***A***_zz_ component of an exchangeable anisotropic α-H proton.

**Figure 1:**
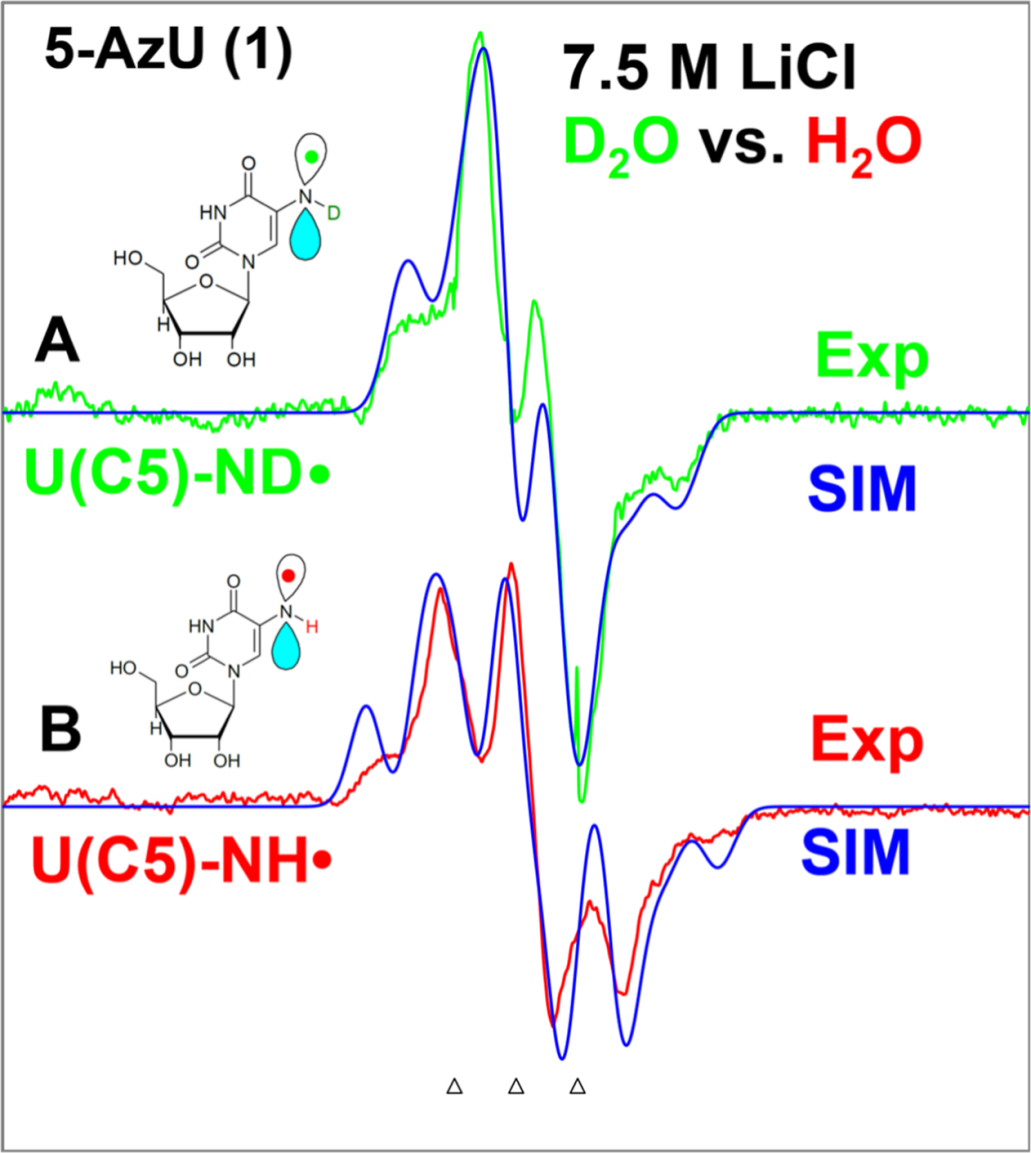
First derivative 77 K EPR spectrum of the aminyl radical (U­(C5)–ND^•^/(U­(C5)–NH^•^)) formed via radiation-produced one-electron attachment by γ-irradiation (absorbed dose = 600 Gy at 77 K) of **1** or 5-AzU (A) (U­(C5)–ND^•^) (green) in 7.5 M LiCl (D_2_O) and (B) (U­(C5)–NH^•^) (red) in 7.5 M LiCl (H_2_O). The simulated spectra (blue) (for simulation parameters, see text below) are superimposed on the top of the experimentally recorded spectrum. The green and red spectra are recorded at 77 K. The three reference markers in this figure and in subsequent figures are Fremy’s salt resonances with the central marker at *g* = 2.0056 and each of the three markers is separated from one another by 13.09 G.^3,8,10,11,46–49,52,53^

In our previous work on RNH^•^ identification from matched samples of 2′-azido-2′-deoxyuridine (2′-AzdU) in H_2_O and D_2_O glasses, the A_∥_ (i.e., the A_*zz*_) component ca. 22 G due to the α-N–H proton of the aminyl radical in H_2_O (U­(C2′)–NH^•^) is lost in the D_2_O glasses as a deuteron coupling is only 15% (1/6.514) that of a proton in the same environment.^49^ Therefore, we assign that the spectra presented in Figure 1 are due to the π-type aminyl radical, RNH^•^. The additional 17 G proton hyperfine coupling in the aminyl radical (U­(C5)–NH^•^) (red, Figure 1B) is present only in the H_2_O glasses and is absent in the D_2_O glasses (green, Figure 1A). These results establish that the solvent (H_2_O) is the source of the proton involved in the formation of the aminyl radical (U­(C5)–NH^•^) via protonation of the nitrene anion radical (U­(C5)–N^•–^). This additional hyperfine coupling of 17 G is the ***A***_***zz***_ component of the N–H (α-H) coupling that contributes to the outer line components of the EPR spectrum and adds to the ***A***_***zz***_ component of the anisotropic hyperfine coupling of the axially symmetric N-atom of U­(C5)–NH^•^. Thus, from the experimentally recorded spectrum (red) of U­(C5)–NH^•^ (Figure 1B), only the ***A***_***zz***_ component of the Ν–Η–(α-H) hyperfine coupling could be determined by experiment.

To further confirm this assignment, the green spectrum of U­(C5)–ND^•^ in Figure 1A and the red spectrum of U­(C5)–NH^•^ in Figure 1B were simulated (blue) using the following parameters. For U­(C5)–ND^•^, the simulation parameters are: C6–H HFCC (17.0, 6.0, 13.6) G, radical site anisotropic axially symmetric N HFCC (0, 0, 20 G) G, N–D (for the exchangeable α-H = 4.0, 1.0, 2.5) G, ***g***_***xx***_, ***g***_***yy***_, ***g***_***zz***_ = (2.0043, 2.0043, 2.0020), a mixed (Lorentzian/Gaussian = 1.0) line shape, and an isotropic line width of 9 G. For U­(C5)–NH^•^, the simulation parameters are: C6–H HFCC (17.0, 6.0, 13.6) G, radical site anisotropic axially symmetric N HFCC (0, 0, 20 G) G, N–H (for the exchangeable α-H = 25.0, 6.0, 17.0) G, ***g***_***xx***_, ***g***_***yy***_, ***g***_***zz***_ = (2.0043, 2.0043, 2.0020), a mixed (Lorentzian/Gaussian = 1.0) line shape, and an isotropic line width of 9 G. The simulated spectra match well with the experimentally observed spectra. Thus, our EPR results unequivocally establish the formation of U­(C5)–ND^•^/U­(C5)–NH^•^ from **1** via reduction (Scheme 2).

**Scheme 2:**
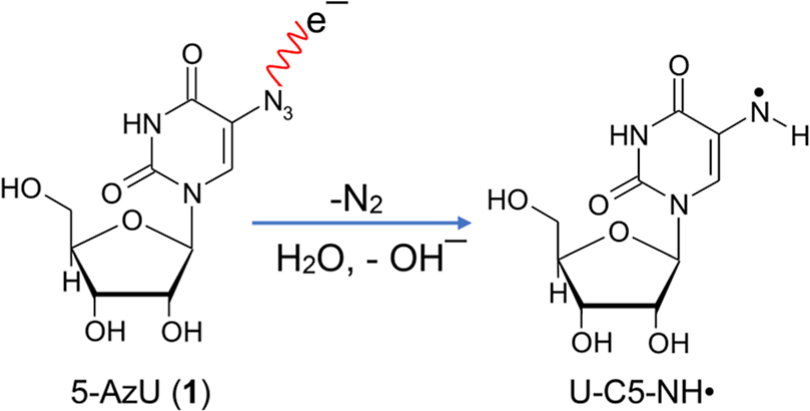
Representation of the Reaction Scheme Showing the Formation of the π-Aminyl Radical, U–U­(C5)–NH^•^, from **1**

We note that the radical site anisotropic axially symmetric N ***A***_***zz***_ HFCC value (ca. 20 G) of U­(C5)–ND^•^/U­(C5)–NH^•^ observed in **1** is approximately half of the corresponding radical site axially symmetric N ***A***_***zz***_ HFCC value (ca. 40.5 to 43 G) reported for RNH^•^ from the azidoPyr nucleosides and azidopentoses. In those systems, the radical site N-atom is either isolated (attached directly to the sugar moiety) or is not directly bonded to the aromatic base-ring (i.e., separated by a –CH_2_– group).^3,8,10,11,46–49^ The anisotropic *g*-values ***g***_***xx***_, ***g***_***yy***_, ***g***_***zz***_ = (2.0043, 2.0043, 2.0020) match well with those reported for RNH^•^.^3,8,10,11,46–49^ The theoretically obtained radical site anisotropic axially symmetric N ***A***_***zz***_ HFCC value (24.1 G, see Supporting Information) of U­(C5)–ND^•^/U­(C5)–NH^•^ found in **1** is close to the experimentally obtained value (ca. 20 G), further supporting our RNH^•^ assignment. Together, all of these results confirm that the U­(C5)–ND^•^/U­(C5)–NH^•^ found in **1** exhibits a π-type nature.

#### 3.2.2 EPR Spectral Studies of One-Electron Addition to 6-Azidouridine (**2**, 6-AzU) Forming an Iminyl σ-Radical, R=N^•^

First derivative EPR spectra of the sample of **2** used in Figure A were annealed to (B) ca. 145 K and subsequently to (C) ca. 155 K. EPR spectra of one-electron oxidized 1-methylcytosine at pH ca. 9.5 (green) and at pH ca. 8 (brown; taken from ref 52) were placed below these spectra. All of the EPR spectra were recorded at 77 K.

The first derivative 77 K EPR spectrum due to radiation (77 K)-produced *e*_pre_^–^ attachment to **2** (blue) in homogeneous glassy (7.5 M LiBr/D_2_O) solutions is shown in Figure 2A. In a LiBr glassy system, Br_2_^•–^ is formed by scavenging of radiation-produced holes in the matrix (7.5 M LiBr); the matrix radical, Br_2_^•–^, does not have line components at the *g* = 2 region.^10^ Thus, unlike LiCl glassy systems in previous studies^3,8,11,46–49^ and this work (Figure 1), no spectral subtraction was required for LiBr glassy samples. The total spectral width, hyperfine splittings, line intensities, and *g*-values at the center of this spectrum are quite similar to that of the previously reported EPR spectrum (purple) of the 1-methylcytosine iminyl σ-radical that was formed in glassy samples (2 mg/mL) of 1-methylcytosine (1-MeCyt) in 7.5 M LiCl/D_2_O after one-electron oxidation of the cytosine base by Cl_2_^•–^.^52,53^ Thus, similarities between these two spectra establish that the blue spectrum is due to two nitrogen HFCC values—one radical site axially symmetric anisotropic N-atom HFCC (0, 0, 40) G and one nearly isotropic N-atom HFCC (9.5, 9.5, 10.2) G along with ***g***_***xx***_, ***g***_***yy***_, ***g***_***zz***_ = (2.0043, 2.0043, 2.0023).^52,53^ Therefore, the blue spectrum obtained from **2** is assigned to the iminyl σ-radical (U–C6=N^•^ (R=N^•^, Scheme 3)). This blue spectrum due to U–C6=N^•^ matches well with recently reported spectra of iminyl σ-radical facilely formed from oxime esters of dCyd (2′-deoxycytidine) via DEA.^53^

**Figure 2:**
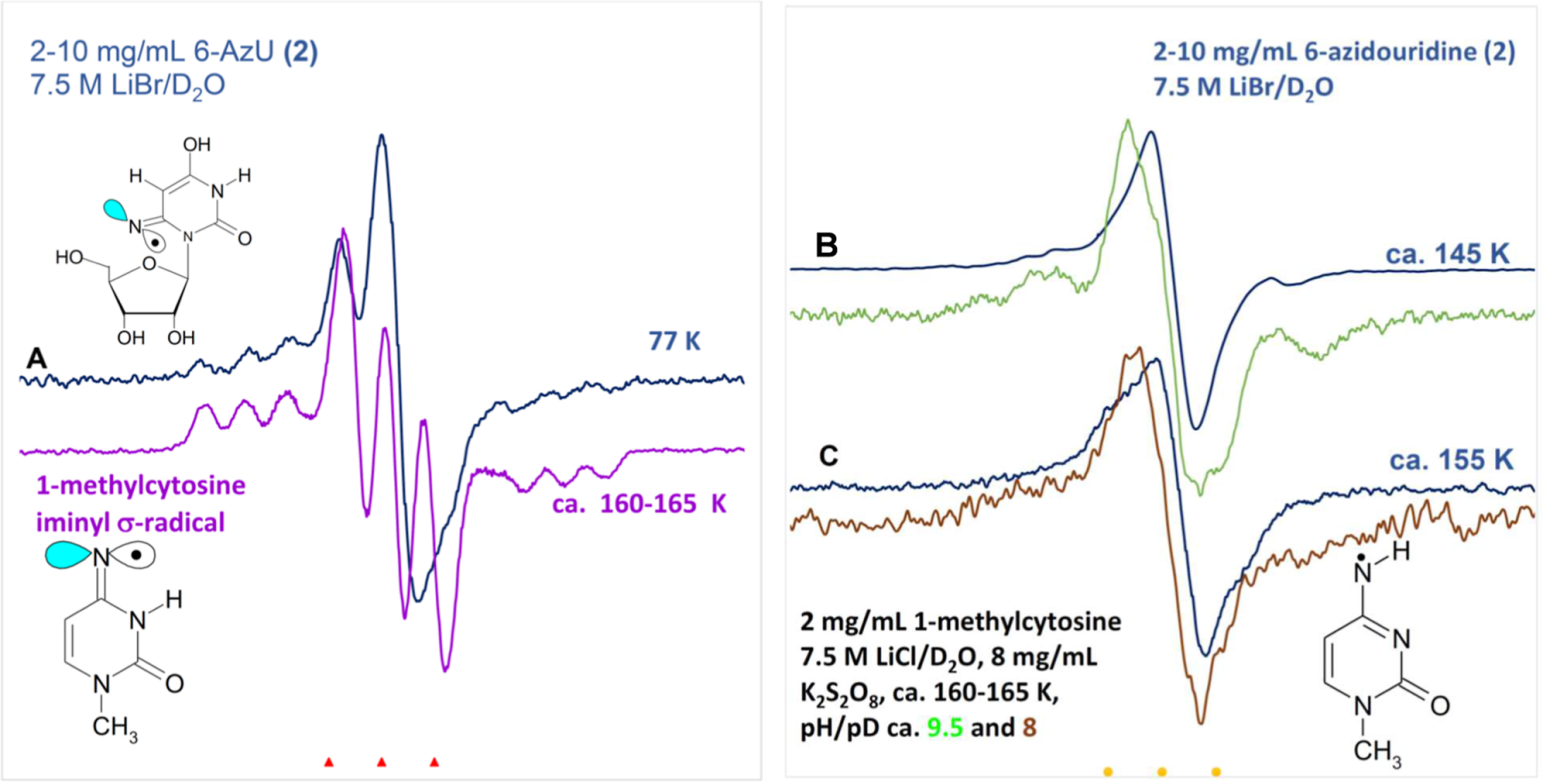
First derivative EPR spectra after one-electron addition to **2** (2.0–10.0 mg/mL, blue) at (A) 77 K in homogeneous glassy (7.5 M LiBr/D_2_O) solution. Electrons were generated by γ-irradiation (absorbed dose = 600 Gy at 77 K) of the glassy solution. The EPR spectrum (purple), taken from ref 52, shown below is that of the 1-methylcytosine iminyl σ-radical formed by one-electron oxidation of 1-methylcytosine by Cl_2_^•–^ (see ref 52 for details).

**Scheme 3:**
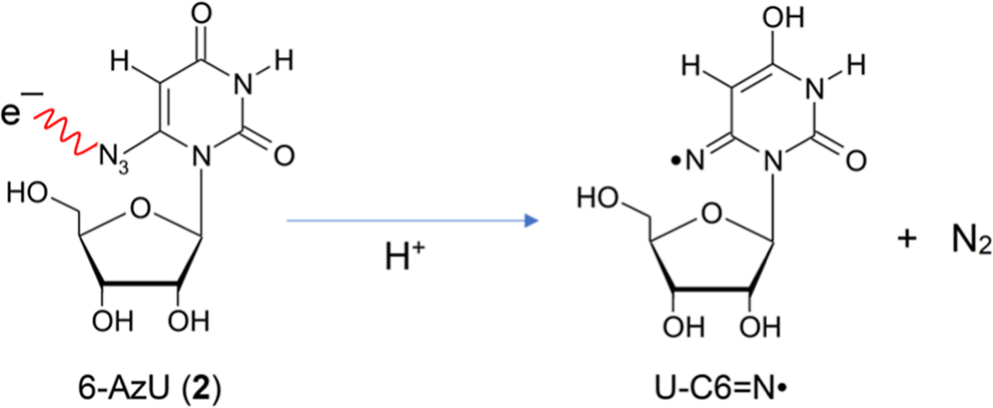
Representation of the Reaction Scheme Showing the Formation of the Iminyl σ-Radical, U–C6=N^•^, from **2**

The theoretically obtained radical site anisotropic axially symmetric N ***A***_***zz***_ HFCC value is shown (38.8 G, see the Supporting Information). Also, the theoretically calculated HFCCs of the nearly isotropic N-atom are ((6.6, 7.1, 9.7) G (see the Supporting Information)). These close agreements of experimental and theoretical HFCC values provide additional support to our U–C6=N^•^ assignment.

These data also establish that: (a) one-electron reduction of **2** leads to the facile formation of U–C6=N^•^ via DEA even at 77 K and (b) the electron affinity of the N_3_ group is higher than that of the pyrimidine base, uracil. This observation supports the earlier work with AZT where we show that the electron affinity of the N_3_ group is higher than the pyrimidine base, thymine.^48^ Scheme 3 illustrates the facile formation of the iminyl σ-radical, U–C6=N^•^, from **2** via DEA. Consistent with the results from **1** (Figure 1 and Scheme 2) and previous observations of NCR formation from azido-DNA models,^3,8,10,11,46–49^ neither the TNI (the azide anion radical, R–N_3_^•–^ or **2** ^•–^) nor the RN^•–^ species formed after dissociation of N_2_ from the TNI were detected by EPR in the homogeneous glassy solution, even at a liquid nitrogen temperature (77 K).

The sample of **2** was further annealed to ca. 145 K and to ca. 155 K to investigate the fate of the U–C6=N^•^. The corresponding EPR spectra (blue) are shown in Figure 2B,C. For comparison, the EPR spectra obtained from previous study on the effect of pH (pH ca. 9.5 (green) and pH ca. 8 (brown)) on the iminyl σ-radical formation from one-electron oxidized 1-MeCyt samples^52^ are presented below these spectra. The brown spectrum (pH ca. 8) in Figure 2C was assigned to the π-type aminyl radical, C4­(N–H)^•^. The green spectrum in Figure 2B was found to be a combination of ca. 70% of the 1-methylcytosine iminyl σ-radical (purple spectrum in Figure 2A) and of ca. 30% of C4­(N–H)^•^ (brown spectrum in Figure 2C). The similarities of these spectra indicate that the blue spectrum in Figure 2C is predominantly due to the protonated form (U–C6–NH^•^, Schemes 3 and 5) observed at 77 K. Since the pH of the 7.5 M LiBr glass is ca. 5 and due to the large abundance of protons in the surrounding environment, conversion of the U–C6=N^•^ to U–C6–NH^•^ is rapid. The indication of this conversion is supported by pulse radiolysis (see the section entitled “Pulse Radiolysis of **2**” and Scheme 5). Therefore, the blue spectrum shown in Figure 2B is not due to a single radical species; rather, it is an overlap of the individual spectrum of U–C6=N^•^ and that of U–C6–NH^•^ in varying amounts. However, the blue spectrum shown in Figure 2C is predominantly due to U–C6–NHring at 77 K with subsequent facile

#### 3.2.3 EPR Spectral Studies of One-Electron Addition to the **3** (4-Azidocytidine Nucleoside Analog) and **4** (Tetrazole Analog of Cytidine) Forming the Azide Anion Radical, RN_3_^•–^

Following the reactions shown in Scheme 3, NCR formation is expected via e_pre_^–^ attachment to **3** (4-azidocytidine nucleoside analog) and **4** (tetrazole analog of cytidine). EPR spectral studies are employed to characterize the type of NCR formation in these compounds.

EPR spectra of the radicals formed from matched γ-irradiated (absorbed dose = 600 Gy at 77 K) 7.5 M LiCl/D_2_O homogeneous glassy samples (concentration = 2–3 mg/mL) of **3** (pink) and **4** (green) were acquired. The EPR spectra were recorded at 77 K just after irradiation and subsequently by gradually warming these samples to ca. 145 K (Figure 3A,B).

**Figure 3:**
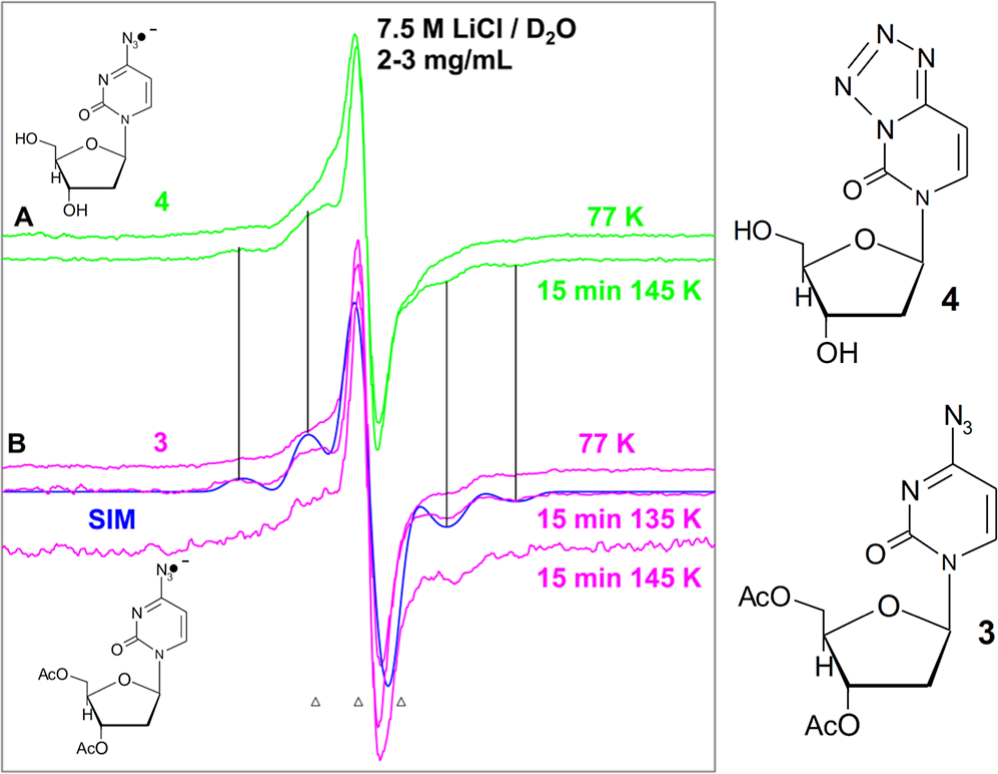
First derivative EPR spectra obtained from matched homogeneous glassy samples (2–3 mg/mL in 7.5 M LiCl/D_2_O (pH/pD ca. 5)) of **4** (tetrazole analog of cytidine) (green) and of **3** (4-azidocytidine nucleoside analog) (pink). The first spectrum in (A,B) shows RN_3_^•–^ formation via radiation-produced (absorbed dose = 600 Gy) e_pre_^–^ attachment at 77 K in the dark. Other spectra in (A,B) were obtained after warming or annealing the samples for 15 min in the dark at ca. 135 K and at ca. 145 K. The simulated spectrum (blue) is shown in (B) and for simulation parameters, refer to the text. Similarities of the ***A***_***zz***_ HFCC in the spectra of both compounds are shown by vertical solid lines. All spectra were recorded at 77 K.

It is evident from the EPR spectra that the center of the spectra, line shape, and overall hyperfine splitting (i.e., total spectral width) did not change before (at 77 K) and after warming (at ca. 145 K) in the dark. The spectra in Figure 3A,B are matched with a simulated spectrum (Figure 3B, blue) that has been obtained by employing a mixed (Lorentzian/Gaussian = 10) line shape, two axially symmetric anisotropic ^14^N (nuclear spin = 1) HFCC values of (22.0, 0, 0) G and (20, 0, 0) G, ***g***_***zz***_, ***g***_***xx***_, ***g***_***yy***_ (2.0017, 2.0041, 2.0041) along with an isotropic Gaussian line-width of 6 G. Even the 77 K EPR spectrum obtained after subsequent photoexcitation of sample from **3** at 145 K for 45 min in the dark remains unchanged. These results establish that the same radical is formed in the base moiety (see Figure 3 and Scheme 4) via the reaction of radiation-produced e_pre_^–^ with both compounds in the homogeneous glassy system;^3,8,10,11,46–49,52,53^ the substitution in the sugar moiety (OH or −OAc) does not affect the base radical formation.

**Scheme 4:**
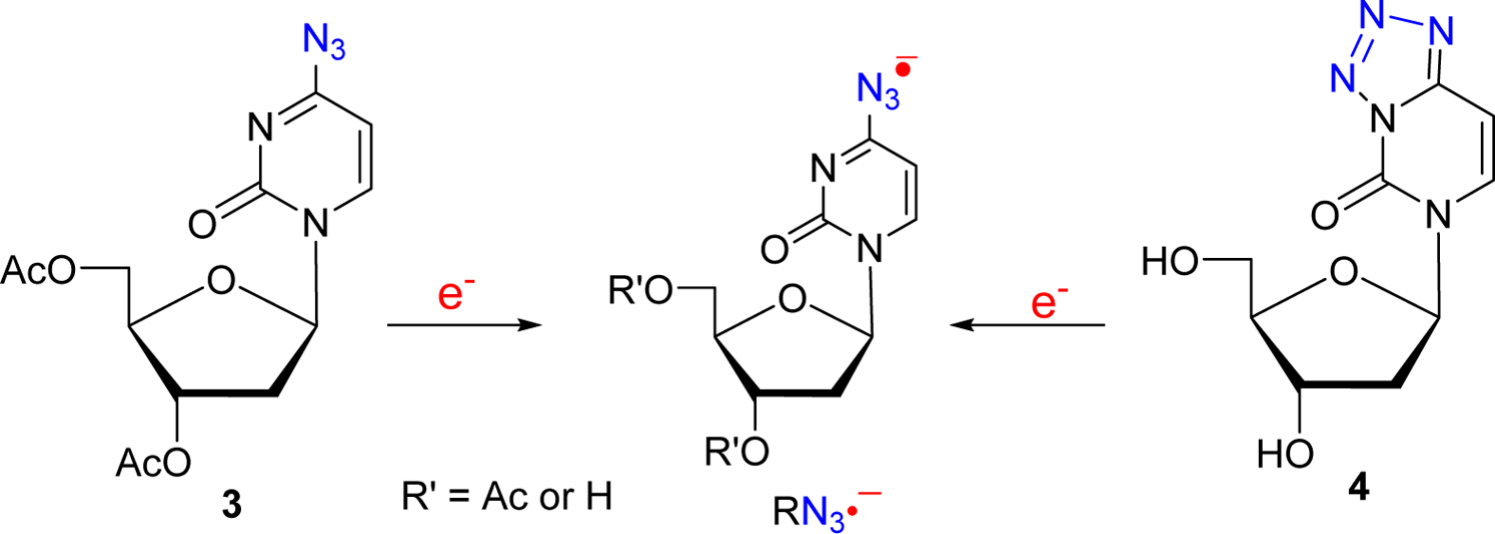
Electron-Mediated RN_3_^•–^ Formation from **3** and **4**

The spectra shown in Figure 3 are different from those reported for aminyl radicals and iminyl radicals in previous studies^3,8,11,46–49,52,53^ and in this work (Figures 1 and 2). We assign the experimentally obtained spectra and the simulated spectrum presented in Figure 3 to the TNI, azide anion radical, RN_3_^•–^. This assignment is supported by theoretical calculations (see the theory section and Supporting Information).

These EPR spectral results further show that: (a) in **4**, RN_3_^•–^ formation occurs via DEA; the radiation-produced e_pre_^–^ attaches to the tetrazole ring at 77 K with subsequent facile breakage of the N-N bond at ring position 3 in the anion radical also at 77 K (Scheme 4). (b) Under the experimental conditions of EPR spectroscopic investigations (homogeneous glassy solutions at low temperatures (77 K to ca. 145 K)), RN_3_^•–^ from these compounds do not convert to the expected neutral π-aminyl radical, C­(N4–H)^•^ ^52^ or iminyl σ-radical (Figure 2),^52,53^ as expected from earlier studies with azido-DNA models.^3,8,10,11,46–49^

### 3.3 Theoretical Calculations

The EPR studies established that the radicals formed via reactions of e_pre_^–^ with **3** and **4** are identical base radicals. Moreover, the nature of the sugar moiety (ribose or deoxyribose) does not affect their formation. We therefore performed theoretical calculations by employing the simpler model compound, 4-azido-1-methyl cytosine, of **3** and **4** to check the influence of the N3 proton on the HFCC values.

SPARTAN 20 (see the Supporting Information) was used to build molecules and anion radicals for the in silico study. Subsequently, the Gaussian’09 suit of programs was employed on these molecules for high-level computational quantum chemistry calculations. These theoretical calculations (Figure 4) predict a particular conformation of the TNI, (azide anion radical, RN_3_^•–^), which gives rise to two high anisotropic N-couplings (***A***_***zz***_ = 26.5 and 35.4 G respectively, see the Supporting Information) compared to the experimentally observed ***A***_***zz***_ values of anisotropic N-couplings (***A***_***zz***_ = 22 G, and 20 G) while ***A***_***xx***_ and ***A***_***yy***_ are too small to resolve for **3** and **4** (see the discussion of the results for Figure 3 above). We also calculated the HFCC values for the same RN_3_^•–^ in 4-azidocytidine and obtained similar values (***A***_***zz***_ = 26.6 and 35.6 G respectively, see the Supporting Information). These results strongly supported the experimental results (Figure 3 and Scheme 4) and the radical assignments.

**Figure 4:**
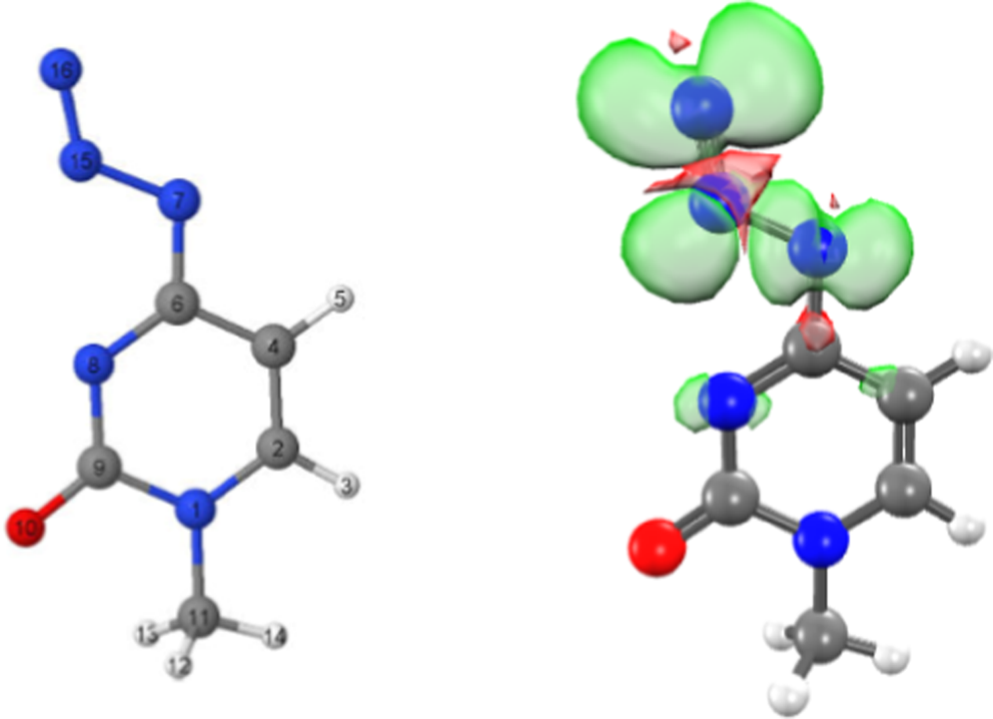
Azide anion radical (RN_3_^•–^) in 4-azido-1-methylcytosine was geometry-optimized employing the DFT/B3LYP/6-31G** method. Spin density distributions and HFCC were calculated employing the geometry-optimized structure of the RN_3_^•–^ using the DFT/B3LYP/6-31G** method considering the full solvation (IEF-PCM) under the Gaussian’09 suit of programs (see the Supporting Information) following previous works.^3,8,11,46–49,52,53^

### 3.4 Pulse Radiolysis

Using different concentrations (0.2, 0.5, and 1 mM) of **1** and **2** at room temperature, we conducted the time-resolved radiolysis of aqueous solutions in the spectral range of 280–700 nm at different timescales. The solutions contained 0.2 M *tert*-butanol to scavenge the hydrogen atom (H^•^) and hydroxyl radicals (^•^OH) produced via water radiolysis, following an established protocol.^9,10,54^ During pulse radiolysis, the solutions were continuously deoxygenated by bubbling with argon gas to minimize the interference of oxygen in subsequent reactions. Under these conditions, only the hydrated (aqueous or fully solvated) electron, e_aq_®, reacted with the solute (**1** or **2**).

The samples (at pH 6.8) were then pumped through flow quartz cells with an optical path length of 0.5 cm throughout the experiment. This continuous flow ensured a consistently fresh solution for optimal interaction during the initial radiolysis process and for the subsequent identification of solute radicals formed from the reactions of e_aq_® with **1** or **2**.

Figure 5A presents the absorption spectrum of the transient species formed via the reaction of e_aq_® with **1**. This spectrum was obtained through Bayesian analysis of the full spectrokinetics data matrix. The transient species is characterized by an absorption maximum at 330 nm and a broad absorption band spanning from λ ∼ 400 to λ ∼ 500 nm, peaking around λ ∼ 450 nm. Beyond λ ∼ 550 nm, the absorbance of the transient species becomes negligible or shows no experimentally observable absorbance.

**Figure 5:**
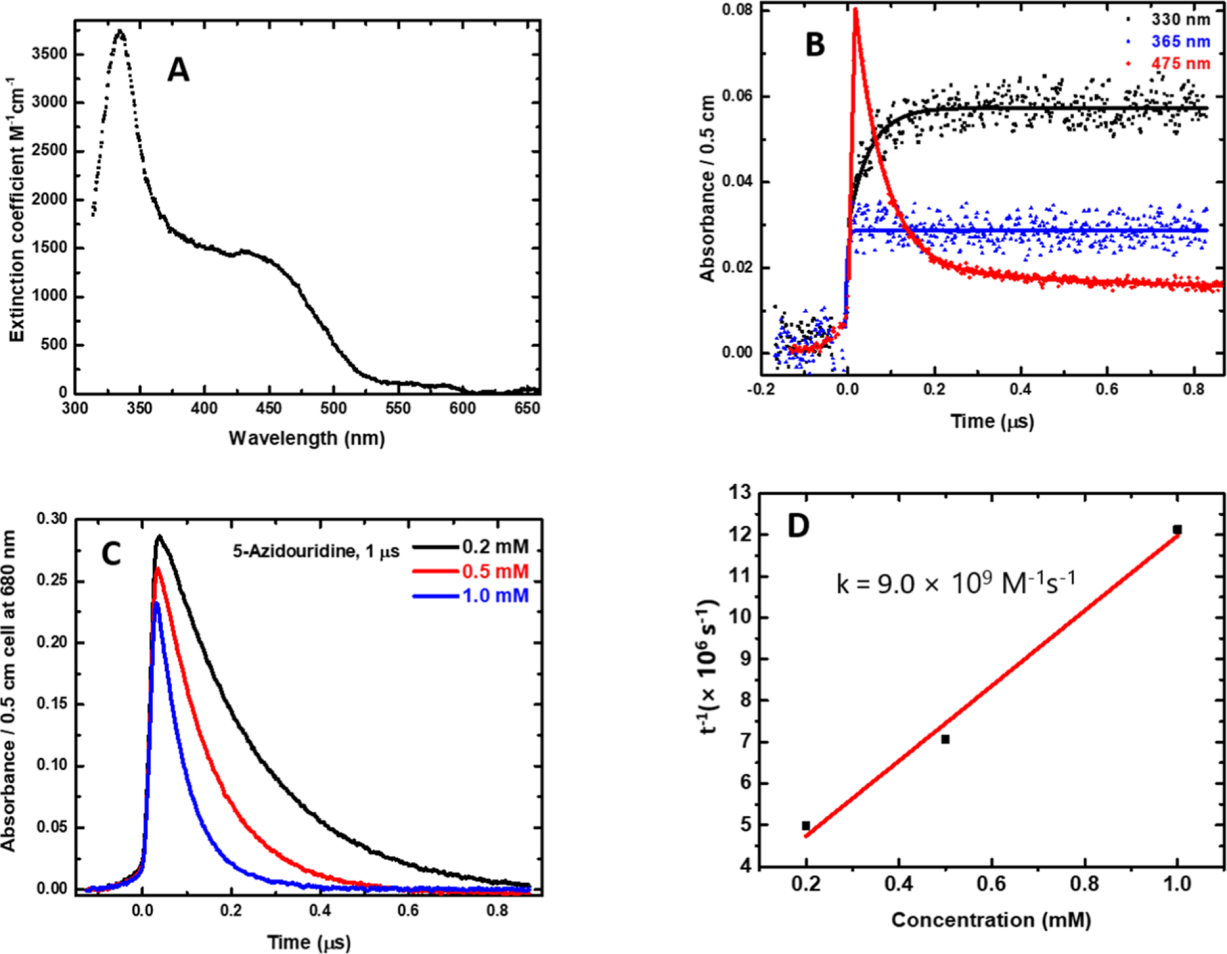
Pulse radiolysis of an Ar-saturated solution of 1 mM of **1** in the presence of 0.2 M *tert*-butanol at room temperature. (A) The absorbance spectrum of U­(C5)–NH^•^ obtained by Bayesian analysis of a full spectro-kinetic data matrix. (B) Kinetics of U­(C5)–NH^•^ formed were observed at 330 nm, 365 nm, and 475 nm. (C) The kinetics observed at 680 nm for three different concentrations (0.2, 0.5, and 1 mM). (D) The rate constant of the reaction of e_aq_® with **1**. The absolute absorption spectrum of U­(C5)–NH^•^ shown in (A) was obtained via the reduction of **1** using the isosbestic point at 365 nm.

Based upon EPR spectral assignment of U­(C5)–ND^•^/U­(C5)–NH^•^ from **1** (Figure 1, this work), and supported by EPR and pulse radiolysis along with theoretically calculated spectra of U–C5–CH_2_NH^•^ from 5-azidomethyl-2′-deoxyuridine,^10,11^ as well as reported absorption spectrum at 77 K of the aminyl radical at the exocyclic aminyl nitrogen (N2) from 1-methylguansoine,^55^ we assign the absorption spectrum reported in Figure 5A to U­(C5)–NH^•^.

The kinetics of U­(C5)–NH^•^ absorbing in the UV region demonstrate wavelength-dependent time-traces within a 1 μs time scale (Figure 5B). The kinetics observed at 330 and 475 nm are found to be distinct. An isosbestic point at 365 nm, due to the reaction between e_aq_® and **1**, is observed (Figure 5B). From this isosbestic point, we deduce that the extinction coefficient of the transient species at 365 nm is about 1870.40 M^–1^ cm^–1^. We reported an extinction coefficient for U–C5–CH_2_NH^•^ from 5-azidomethyl-2′-deoxyuridine as 1180 M^–1^ cm^–1^.^10^ Following EPR studies on U­(C5)–NH^•^ from **1**, we attribute this increase of the extinction coefficient to the π-type nature of the aminyl radical, in which the radical site anisotropic axially symmetric N-atom is directly connected to the pyrimidine ring, in contrast to the –CH_2_-bridged structure of U–C5–CH_2_NH^•^ from 5-azidomethyl-2′-deoxyuridine.

At 680 nm, the kinetics of the decay of e_aq_® correlated well with the increasing concentrations (0.2, 0.5, and 1 mM) of **1** in solution. For illustration, the decay kinetics of e_aq_® at 680 nm are presented in Figure 5C. Using these data, the bimolecular rate constant for the reaction of e_aq_® with **1** was determined to be 9.0 × 10^9^ M^–1^ s^–1^ (Figure 5D), indicating a diffusion-controlled reaction.

In conclusion, these results demonstrate that the DEA-mediated RNH^•^ (U­(C5)–ND^•^/U­(C5)–NH^•^) formation from **1** proceeds via the same pathway as observed in previous studies on azidoDNA-models.^3,8,10,11,46–49^ Neither the TNI (the azide anion radical, R–N_3_^•–^ or **1** ^•–^) nor the RN^•–^ formed via dissociation of N_2_ from the TNI was detected using EPR in the homogeneous glassy solution at a liquid nitrogen temperature (77 K) or using pulse radiolysis in an aqueous solution at room temperature.

#### 3.4.1 Pulse Radiolysis of **2**

The reaction of e_aq_® with **2** was investigated by employing pulse radiolysis, as described previously for **1**. A transient species was observed to form at a diffusion-controlled rate within 1 μs, with its absorption spectrum (shown in red) presented in the left panel of Figure 6. This transient species exhibits a broad absorption spectrum spanning from λ ∼ 320 to λ ∼ 700 nm, with two prominent bands peaking at λ ∼ 390 and λ ∼ 550 nm as shown in Figure 6A. We have presented the SPARTAN 20 (DFT/B3LYP/6-31+G*, polar solvent)-calculated optimized structures and the spin density distributions of the σ-type 6-iminyl radical (U–C6=N^•^ and in Figure S1d). The spin density distribution follows the SOMO (singly occupied molecular orbital) of the radical. This absorption spectrum closely resembles those previously reported for NCR, having the radical site at N1 in the ring from pyrimidines (e.g., thymine, uracil), generated via one-electron oxidation by SO_4_^•–^.^56,57^ Based on these findings and the EPR spectral data for **2** shown in Figure 2, we assign this red spectrum to that of the iminyl σ-radical, U–C6=N^•^ (Scheme 3).

**Figure 6:**
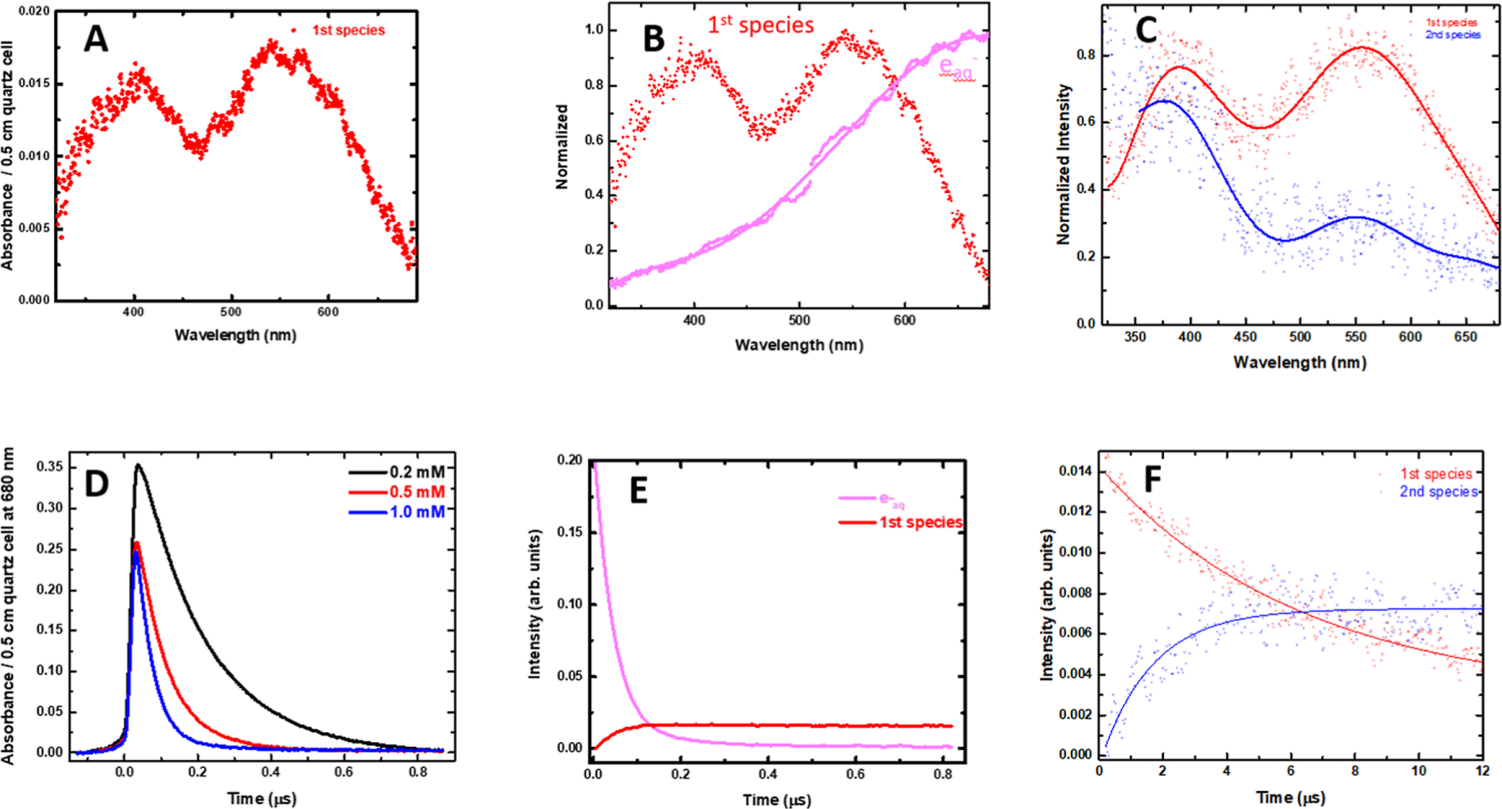
Pulse radiolysis of Ar-saturated solution of 1 mM of **2** in the presence of 0.2 M *tert*-butanol at room temperature. Left panel: the experimentally measured transient absorption spectrum (Figure 6A) with the kinetics of the decay of e_aq_® at different concentrations of **2** at 1 μs (Figure 6D). Middle panel: Transient absorption spectrum of the first species and e_aq_® (Figure 6B) obtained by a Bayesian analysis of the full spectro-kinetics data matrix and the corresponding kinetic formation of the first species produced by the reaction of e_aq_® (Figure 6E). Right panel: Transient absorption spectrum of the first species, U–C6=N^•^ (red) and the formation of the second species, U–C6–NH^•^ (blue) (Figure 6C), observed by employing pulse radiolysis of aqueous solution of **2** at ≤20 μs. Figure 6F presents the corresponding kinetics of the decay of U–C6=N^•^ and formation of U–C6–NH^•^ at ≤20 μs.

At 680 nm, the kinetics of the decay of e_aq_® correlated well with increasing concentrations (0.2, 0.5, and 1 mM) of **2** in solution, as illustrated in Figure 6D. Using these kinetics, the bimolecular rate constant of the reaction of e_aq_® with **2**, forming U–C6=N^•^, is determined as 1.0 × 10^10^ M^–1^ s^–1^. At this rate, the reaction is considered a diffusion-controlled one.

Subsequently, in the 20 μs time scale, U–C6=N^•^ decays by first-order kinetics to form another transient species (blue spectrum, Figure 6C, right panel). This absorption spectrum shows a less intense absorption band around 550 nm along with a blue-shifted absorption band at 375 nm in comparison to the two bands observed in the spectrum of U–C6=N^•^ (Figure 6A, left panel). Based on the reported absorption spectra of NCRs due to (i) azide radical (N_3_^•^)-mediated one-electron oxidation of tryptophan^58–60^ and (ii) N_3_^•^-mediated one-electron oxidation of the bisbenzimidazole DNA-ligand, Hoechst 33258 and 33342,^61^ we assign this blue spectrum (right panel, Figure 6C) to the neutral aminyl radical, U–C6–NH^•^, formed via water (solvent)-assisted protonation of U–C6=N^•^ (Scheme 5) followed by protonation of the N-atom at C6. Figure 6F presents the corresponding kinetics of the decay of the first species (U–C6=N^•^) and the formation of the second species (U–C6=N^•^) at ≤20 μs. EPR studies of one-electron-oxidized 1-methylcytosine and derivatives also reported pH-dependent conversion of R=N^•^ to R–NH^•^.^52^ As this reaction follows first-order kinetics and taking into account the time scale (≤20 μs), the rate constant of this conversion (U–C6=N^•^ to U–C6–NH^•^, Scheme 5) is = (0.693/(≤20 μs)) = ≤3.5 × 10^4^ s^–1^. This rate appears to be quite reasonable under neutral (pH ca. 7) conditions; under acidic conditions, this rate is much faster (e.g., see Figure 2 and its discussion).

**Scheme 5:**
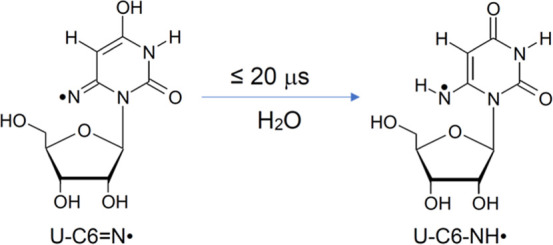
Solvent (Water)-Assisted Conversion of U–C6=N^•^ to U–C6–NH^•^

### 3.5 Ion Chromatography

To elucidate whether the radiolysis of the molecules reported in this work induces the release of N_3_® in solutions, analysis of the irradiated solutions of **1** was carried out by ion chromatography as an example.

At first, the ion chromatography measurements of aqueous solutions containing different concentrations of N_3_® were carried out to establish the intensity–concentration curve and the retention time of N_3_® in aqueous solution.

With a flow rate of 1.20 mL/min, the N_3_® was determined in 8.4 min using an IC SI-90 4E column.

For the gamma-irradiated solutions of **1**, we employed experimental conditions like those used for pulse radiolysis. Specifically, the samples of **1** contained 0.2 M *tert*-butanol as a scavenger of ^•^OH, and the solutions were saturated with argon to eliminate oxygen and create a reductive environment that favors the e_aq_® reaction with **1**. Under these conditions, the chromatograms displayed no peak of N_3_®, indicating no N_3_® release (see the Supporting Information, Section 4). This result suggests that DEA to the azide-modified DNA model compounds does not lead to a measurable release of N_3_® when a radiation-produced electron (here it is e_aq_® in solution) is the primary reactive species.^3,8,10,11,46–49^ We also note here that N_2_ extrusion has been reported in simple azides (methyl and ethyl azide as well as aromatic azides, e.g., azidobenzene, nitroazidobenzene).^62^ However, only in one case, 6-azidomethyluridine, N_3_® loss through DEA has been observed to date.^10^

## 4 Conclusions

A combination of synthesis, EPR studies in homogeneous glassy aqueous solution at low temperature, pulse radiolysis in aqueous solutions at room temperature, DFT calculations, and analytical technique (notably ion chromatography) led to the following salient points in this work:

a EPR and DFT studies establish that the addition of an N_3_ group to the 4-(**3** and **4**) position of the Pyr base moiety leads to the formation of the TNI, a stable azide anion radical, Pyr–N_3_^•–^, after electron attachment. These studies further establish that the subsequent step (i.e., dissociation) of the DEA pathway does not happen in this case.

On the other hand, a combination of EPR, Pulse radiolysis, and DFT studies unequivocally shows that the addition of an N_3_ group to the 5-(**1**) and 6-(**2**) positions of the Pyr base moiety leads to the formation of an aminyl radical, PyrNH^•^ (from **1**), and a conjugated iminyl σ-radical, Pyr=N^•^ (from **2**) via DEA. Consequently, the stabilization of NCRs (RN_3_^•–^, RNH^•^, R=N^•^) is dictated by the position of the N_3_ group substitution on the Pyr base moiety. These results along with DFT calculations establish that neither the nature of the sugar moiety (ribose, deoxyribose) nor the substitution (−OAc, –OH) affect the nature of the resulting NCR.

b Ion chromatography of **1** confirmed the DEA pathway that we established through the combination of EPR, pulse radiolysis, and DFT calculations (this work) and previous studies^3,8,10,11,46–49^ with the only exception being 6-azidomethyluridine.^10^
c In some of our previous works on azidonucleosides, the N_3_ group is directly attached to the sugar moiety.^47–49^ We also studied azidonucleosides with the N_3_ group displaced by a –CH_2_– group either to the base-ring^10,11^ or to the sugar moiety.^48,49^ In contrast, this study investigates azidonucleosides with the N_3_ group directly connected to the pyrimidine ring (Scheme 1). This type of structure enables us to highlight the difference in the extent of electron spin density localization at the radical site and, hence, reactivity of the NCR (e.g., R–NH^•^) obtained in this study versus those in previous work.

For example, the radical site anisotropic axially symmetric N ***A***_***zz***_ HFCC value (ca. 20 G) of U­(C5)–ND^•^/U­(C5)–NH^•^ found in **1** is found to be approximately half of that of the corresponding radical site axially symmetric N ***A***_***zz***_ HFCC reported value (ca. ranging from ca. 40.5 to ca. 43 G) of RNH^•^ from the azidopyrimidine nucleosides and azidopentoses.^10,11,47–49^ Thus, the radical site N-atom of RNH^•^ from **1** has ca. 50% less spin density than that of the radical site N-atom of RNH^•^ from the azidopyrimidine nucleosides and azidopentoses.^10,11,47–49^ Since the amount of the spin density on the radical site crucially influences the radical’s reactivity, this work predicts that the NCR obtained from the azidopyrimidine nucleosides with the N_3_ group directly attached to the Pyr base would be less reactive than the corresponding NCR formed from the azidopyrimidine nucleosides in which the N_3_ group is directly attached to the sugar moiety.^47–49^

d EPR and pulse radiolysis identify the formation of an iminyl σ-radical (U-C6=N^•^) in a nucleobase-model system showing near-isotropic β-hyperfine ring nitrogen coupling. In addition, the EPR and pulse radiolysis present the evidence of a solvent (water)-assisted conversion of U–C6=N^•^ to its more stable form (U–C6–NH^•^). Pulse radiolysis allowed us to determine the time scale of this conversion.
e Previous work^10^ established that DEA in 6-azidomethyluridine (a model of 6-azidomethylpyrimidine nucleosides) leads to form the “allylic” UCH_2_^•^ along with N_3_® loss. The spin density in the “allylic” UCH_2_^•^ is highly delocalized. However, the spin density distribution in the iminyl σ-type radical (U–C6=N^•^) formed from **2** is primarily on the radical-site N-atom and not on the aromatic ring due to its σ-type nature (Figure S1).
f This work together with previous studies^3,8,10,11,47–49^ on azidonucleosides established electron-mediated (i.e., via reduction) site-specific formation of oxidative π-type aminyl radical (RNH^•^) and σ-type iminyl radical (R=N^•^). Our earlier findings^52,53^ corroborated by other works in literature^27,28^ have demonstrated that similar NCR (RNH^•^ and R=N^•^) are produced via one-electron oxidation pathways.
g The type of NCR formed could be used to predict the damaging potential of the compound. Previous works on the type of formation of NCR and its reactivity in azidonucleosides so far^3,8,10,11,47–49^ have established that RNH^•^ can oxidize guanine, can abstract H-atom, and can also form tandem lesions by adding to the double bond. R=N^•^ can form tandem lesions but not strand breaks.^53^ However, RN_3_^•–^, if stable to N_2_ loss, can then only donate its extra electron to an electron-accepting species via an electron transfer reaction. This should lead to the formation of the original 4-azidopyrimidine nucleoside and a reduced species. Thus, from the viewpoint of predicting the damaging potential, **1** should be the most damaging and **3** (or **4**) should be the least damaging. We are planning to test this prediction.

## Supplementary Material



## Data Availability

The original data are available for sharing upon request to the corresponding authors by email.

## Notes

Relevant information for the synthesis of compounds **2**, **3**, and **4**; sample preparation and methodologies for EPR studies; methodologies of theoretical calculations; and methodology of the ion chromatography (PDF)
