# Supplementary material for: The Site of Azido Substitution in a Pyrimidine Nucleobase Dictates the Type of Nitrogen-Centered Radical Formed after Dissociative Electron Attachment
Source: J Phys Chem B. 2025 Aug 4;129(32):8115–26. doi: 10.1021/acs.jpcb.5c02751 (PMC12359106; doi:10.1021/acs.jpcb.5c02751)
Supplement: Supplementary file 1 [file jp5c02751_si_001.pdf]

The Site of Azido Substitution in a Pyrimidine Nucleobase Dictates the Type of Nitrogen-Centered Radical Formed after Dissociative Electron Attachment

Daniel Adjei,[b,1] Maria de Cabrera,[a,1] Yahaira Reyes,[a,1] Alexandru Barbolovici,[c] Moaadh Alahmadi,[c] Samuel Ward,[c] Marie-Claude Menet,[d] Philippe Mejanelle,[d] Anil Kumar,[c] Michael D. Sevilla,[c] Stanislaw F. Wnuk,[a,*] Mehran Mostafavi,[b,*] and Amitava Adhikary[e,*]

[a]*Department of Chemistry and Biochemistry, Florida International University, Miami, Florida 33199, United States.*

[b]*Institut de Chimie Physique, UMR 8000 CNRS, Bât. 349，Université Paris-Saclay; 91405 Orsay, Cedex, France.*

[c]*Department of Chemistry, 146 Library Drive, Oakland University, Rochester, Michigan 48309, United States.*

[d]*IUT d'Orsay, 91190 Gif-sur-Yvette, France.*

[e]*Free Radical and Radiation Biology Program, 375 Newton Road, 4216 Medical Education Research Facility, Department of Radiation Oncology, Carver College of Medicine, University of Iowa, Iowa City, Iowa 52242, United States.*

[1] These authors contributed equally and are considered as joint first authors

Corresponding Author: Stanislaw F. Wnuk
Phone: (305) 348-6195 Fax (305) 348-3772 E-mail: wnuk@fiu.edu
ORCID ID: 0000-0002-3111-3919

Corresponding Author: Mehran Mostafavi
E-mail: mehran.mostafavi@universite-paris-saclay.fr
ORCID ID: 0000-0002-4510-8272

Corresponding Author: Amitava Adhikary
Phone: +13193350242 E-mail: amitava-adhikary@uiowa.edu
ORCID ID: 0000-0001-9024-9579

SUPPLEMENTARY INFORMATION

CONTENTS

1. **Chemical Synthesis** S3-S4
2. **EPR Studies** S4-S5
    2.1. Sample preparation S4
    2.2 Methods S5
3. **Theoretical Studies** S5-S17
    3.1. Method of theoretical calculations S5
    3.2. Results/output S6-S21
Figure S1 (SPARTAN) S22
4. Ion Chromatography S23-S24
4. **References** S25-S26

1. Chemical Synthesis

The 4-azido-2-pyrimidinone nucleoside **3** was prepared from the 3',5'-di-O-acetyl protected 4-triazonyl derivative **S1**[1,2] (Scheme S1). Thus, treatment of **S1** with NaN_3/DMF[3] gave low to moderate yield of *3*. *Adding acetic acid to the reaction mixture and using* H_2O/DMF (1:4) as solvent provided **3** in 85% yield. Attempted deprotection of **3** with methanolic ammonia, NaOMe, or NaCN/ethanol resulted in decomposition as observed before in similar azido compounds.[3] Remarkably, deprotection in MeOH saturated with HCl gave deacetylated 4-tetrazolo product **4**[4] quantitatively with ^{1}H NMR showing a deshielding of the H5 and H6 peaks in **4** as compared to **3**.

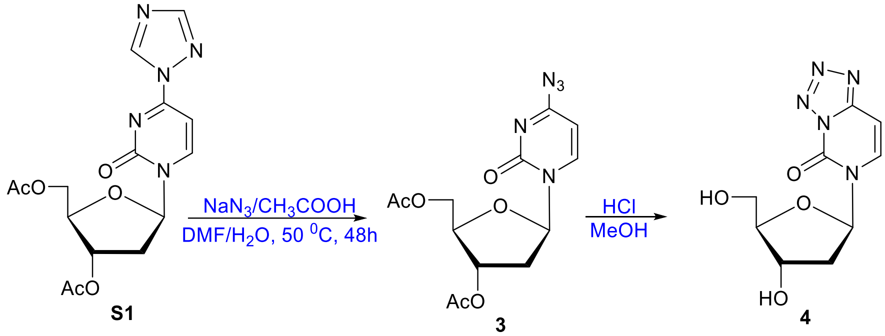

Scheme S1. Synthesis of 4-azido-2-pyrimidinone nucleoside **3** and its cyclic tetrazolo isomer **4**

1-(3,5-Di-*O*-acetyl-2-deoxy-β-D-ribofuranosyl)-4-azidopyrimidin-2(1*H*)-one (3). The 4-triazolo **S1** (250 mg, 0.69 mmol) was dissolved in 4 mL of DMF/H_2O (3:1) and CH_3COOH (500 µL, 525 mg, 8.74 mmol) was added. The NaN_3 (280.4 mg, 4.31 mmol) was dissolved in H_2O (1 mL) and added to the reaction. The mixture was stirred at 50°C for roughly 72 h. Then, the reaction mixture was diluted with EtOAc (20 mL), and washed with $NaHCO_3$, H_2O, and brine. The organic layer was dried over $MgSO_4$ and was evaporated. The residue was column chromatographed (80% EtOAc/Hex) to give **3** (198 mg, 85%): ^{1}H NMR ($CDCl_3$) δ 2.11 (s, 3H, CH_3), 2.16 (s, 3H, CH_3), 2.22-2.31 (m, 1H, H2'), 2.72-2.80 (m, 1H, H2"), 4.33-4.41 (m, 2H, H4', H5'), 4.45 (dd, J = 3.4, 11.4 Hz, 1H, H5"), 5.27-5.31 (m, 1H, H3'), 6.52 (dd, J = 5.6, 8.1 Hz, 1H, H1'), 6.95 (d, J = 7.9 Hz, 1H, H5), 7.77 (d, J = 7.9 Hz, 1H, H6); HRMS (TOF, ESI) *m/z* calcd for $C_{13}H_{16}N_5O_6$ 338.1095 $[M + H]^+$, found 338.1087.

6-(2-Deoxy-β-D-ribofuranosyl)tetrazolo[l,5-*c*]pyrimidin-5(6*H*)-one (4). Compound **3** (50 mg, 0.15 mmol) was dissolved in 2 mL of MeOH and cooled to 0°C, then 2 mL of MeOH saturated with HCl was added. The solution was stirred for 3 h at 0°C. The volatiles were evaporated, and the residue was immediately column chromatographed (5% MeOH/CH_2Cl_2) to give **4**[1] (36.1 mg, 95%): ^{1}H NMR (CD_3OD)

δ 2.43-2.48 (m, 1H, H2'), 2.52-2.55 (m, 1H, H2"), 3.72 (dd, *J* = 3.5, 12.1 Hz, 1H, H5'), 3.87 (dd, *J* = 3.1, 12.1 Hz, 1H, H5"), 4.05-4.10 (m, 1H, H4'), 4.43-4.49 (m, 1H, H3'), 6.52 (t, *J* = 6.5 Hz, 1H, H1'), 7.02 (d, *J* = 7.8 Hz, 1H, H5), 8.33 (d, *J* = 7.9 Hz, 1H, H6); HRMS (TOF, ESI) *m/z* calcd for $C_9H_{12}N_5O_4$ 254.0884 $[M + H]^+$, found 254.0878.

2. EPR Studies

2.1 Sample preparation

Compounds purchased:

Glassy sample preparation:

(i) Preparation of homogeneous solutions: First, homogeneous solution was prepared by dissolving 2 to 10 mg/mL of a compound (e.g., **2**) in either 7.5 M LiCl in D_2O or in H_2O and in LiBr in D_2O. The native pH of 7.5 M LiCl in D_2O or in H_2O is ca. 5 and pH of these solutions was not adjusted.[5-9]

(ii) Preparation of glassy samples and their storage: Homogenous solutions of the compounds were thoroughly bubbled with nitrogen gas. Subsequently, those solutions were immediately drawn into 4 mm Suprasil quartz tubes (Catalog no. 734-PQ-8, WILMAD Glass Co., Inc., Buena, NJ, USA). The quartz tubes containing these solutions were then rapidly immersed in liquid nitrogen (77 K). Owing to rapid cooling at 77 K, the homogeneous liquid solutions formed transparent homogeneous glassy solutions. Homogeneous glassy solutions of these compounds were subsequently γ-irradiated at 77 K and then subjected to progressive annealing experiments during the EPR studies. All glassy samples were stored in the dark at 77 K in Teflon containers.[5-9]

2.2 Methods

γ-Irradiation of glassy samples and their storage: Following our well-established methodology of γ-irradiation of glassy samples of nucleoside-models, the glassy samples were γ (^{60}Co)- irradiated (absorbed dose = 500 Gy; ca. 3-4 h) at 77 K and stored at 77 K in Teflon containers in the dark.[5-15]

Annealing of glassy samples: Following our protocols, A variable temperature assembly that passed liquid nitrogen cooled dry nitrogen gas past a thermister and over the glassy sample was employed for annealing following our previous studies. Stepwise (either 5 K or 10 K step) annealing of each glassy sample was conducted in the range (ca.135 to ca.165) K for 15 min. [5-15]

Electron Paramagnetic Resonance: Following our ongoing studies on DNA and RNA-radicals,[5-15] we used a Varian Century Series X-band (9.3 GHz) EPR spectrometer with an E-4531 dual cavity, 9-inch magnet, and a 200 mW Klystron. For the field calibration, Fremy's salt (g_{center} = 2.0056, $\boldsymbol{A}$(N) = 13.09 G) was employed. All EPR spectra were recorded at 77 K, at 45 dB (6.3 μW) as well as 40 dB (20 μW), at the central field = 3300 G, and at the microwave frequency = 9.3 GHz. Using Fremy's salt field calibration (g_{center} = 2.0056, $\boldsymbol{A}$(N) = 13.09 G) and the central field, the total hyperfine splitting of the experimentally

recorded spectrum, the individual HFCC values, and the *g*-value of the radical (aminyl or iminyl) are determined.

A Bruker EMXPplus-9.5/2.7/P/L X-band continuous wave EPR spectrometer was also used for EPR measurements, and all samples were run under identical conditions (100 kHz field modulation, 320 G scan range centered at 3280 G, 3.2 G modulation amplitude and microwave power of 45 dB (6.3 μW) at 77K).[18]

We note here that recording of EPR spectra at 77 K maximizes the signal height and allows for comparison of signal intensities at the same temperature.

Employing the Bruker programs (WIN-EPR and SimFonia) and following our ongoing studies on DNA and RNA-radicals,[5-17] anisotropic simulations of experimentally recorded EPR spectra of various radicals were carried out using the experimentally obtained hyperfine coupling constant (HFCC) values and *g*-values. The EPR parameters (e.g., HFCC values, linewidth, etc.) were adjusted to obtain the "best fit" simulated spectrum that matched the experimental EPR spectrum well.[5-17] In addition, each EPR spectrum reported in the main manuscript as well as in the supporting information is obtained after subtraction of line components due to $Cl_2^{\bullet -}$.

3. Theoretical Studies

<u>3.1 Method of theoretical calculations</u>:

The geometries of radicals were first optimized as implemented in the Gaussian 09 suit of program using the DFT/B3LYP/6-31G** method.[19,20] Employing optimized geometries of these radicals, spin densities were subsequently plotted; energies of radicals and hyperfine coupling constant (HFCC) values were calculated using DFT/B3LYP/6-31G** method (*vide infra*) in Gaussian 09. Theoretically predicted HFCC values obtained employing B3LYP/6-31G** method agree well with those obtained using experiment.[5-17,21] Jmol molecular modeling freeware was used to plot optimized molecular structures.[22] The isotropic and anisotropic hyperfine couplings contributing to the radicals' EPR spectra are highlighted by yellow.

In this work, we investigated the only base radicals of azido-pyrimidine nucleosides. Therefore, calculations are performed employing azidopyrimidine ribonucleosides with azido substitution at 4-, 5-, and 6- position of the pyrimidine ring.

We employed SPARTAN 20 (DFT/B3LYP/6-31+G*, polar solvent)[23] to calculate structures and the spin density distributions of the geometry-optimized radicals formed from the azidopyrimidine ribonucleosides with azido substitution at 4-, 5-, and 6- position of the pyrimidine ring (Figure S1 on page S18). The geometry optimization was done using the same methodology. These spin density distributions were used in discussion of the EPR results (Figure 2), and in the TOC graphic of this work.

3.2 Results/Output:

B3LYP-PCM/6-31G**

U(C5)-NH• from 5-AzU, **1**

TE = -965.783385878 A.U.

Charge = 0 Multiplicity = 2

Mulliken charges and spin densities:

		1	2
1	O	-0.556649	-0.000171
2	C	0.325996	-0.002069
3	C	0.125275	0.005689
4	C	0.129009	-0.000237
5	C	0.161713	0.000165
6	C	0.046383	0.000087
7	O	-0.539947	0.002518
8	H	0.145761	-0.000262
9	H	0.137418	-0.000484
10	O	-0.568584	0.000092
11	H	0.349141	0.000033
12	H	0.113627	0.000271
13	H	0.130570	-0.000055
14	H	0.116246	0.000008
15	H	0.116384	0.000001
16	O	-0.588043	-0.000001
17	N	-0.526311	0.050958
18	C	0.801013	0.016191
19	O	-0.530775	0.070829
20	N	-0.615831	-0.016321
21	C	0.616692	0.020006
22	O	-0.534183	0.004609
23	C	0.155441	-0.150097
24	C	0.174395	0.455375
25	H	0.313650	0.000373
26	H	0.174330	-0.020315
27	N	-0.599077	0.581903
28	H	0.255482	-0.019068
29	H	0.327981	-0.000007
30	H	0.342890	-0.000018

Sum of Mulliken charges = -0.00000 1.00000

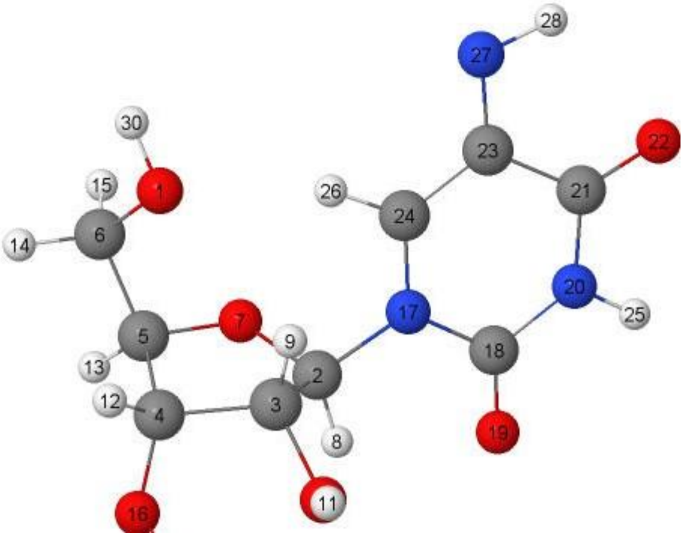

U(C5)-NH• from 5-AzU, **1**

Isotropic Fermi Contact Couplings

	Atom	a.u.	MegaHertz	Gauss	10(-4) cm-
1	O(17)	-0.00100	0.60380	0.21545	0.20141
2	C(13)	-0.00171	-1.92055	-0.68530	-0.64063
3	C(13)	0.00046	0.51219	0.18276	0.17085
4	C(13)	-0.00035	-0.38883	-0.13874	-0.12970
5	C(13)	-0.00007	-0.08033	-0.02866	-0.02679
6	C(13)	0.00005	0.05085	0.01814	0.01696
7	O(17)	0.00185	-1.12371	-0.40097	-0.37483
8	H(1)	-0.00006	-0.26368	-0.09409	-0.08795
9	H(1)	-0.00007	-0.30753	-0.10974	-0.10258
10	O(17)	-0.00023	0.13778	0.04916	0.04596

11	H(1)	0.00006	0.26823	0.09571	0.08947
12	H(1)	0.00016	0.72148	0.25744	0.24066
13	H(1)	-0.00002	-0.08727	-0.03114	-0.02911
14	H(1)	0.00001	0.02387	0.00852	0.00796
15	H(1)	0.00001	0.03522	0.01257	0.01175
16	O(17)	0.00004	-0.02163	-0.00772	-0.00722
17	N(14)	0.00759	2.45102	0.87458	0.81757
18	C(13)	-0.00073	-0.82028	-0.29269	-0.27361
19	O(17)	0.01071	-6.49479	-2.31750	-2.16643
20	N(14)	-0.00467	-1.50876	-0.53836	-0.50327
21	C(13)	0.00287	3.22680	1.15140	1.07634
22	O(17)	0.00029	-0.17750	-0.06334	-0.05921
23	C(13)	-0.03209	-36.07487	-12.87241	-12.03328
24	C(13)	0.04391	49.36414	17.61435	16.46611
25	H(1)	0.00020	0.90622	0.32336	0.30228
26	H(1)	-0.00736	-32.89241	-11.73683	-10.97173
27	N(14)	0.07638	24.67947	8.80625	8.23218
28	H(1)	-0.00850	-37.98250	-13.55310	-12.66960
29	H(1)	-0.00000	-0.01104	-0.00394	-0.00368
30	H(1)	-0.00000	-0.00272	-0.00097	-0.00091

Anisotropic Spin Dipole Couplings in Principal Axis System

Atom		a.u.	Megahertz	Gauss	10(-4) cm-1	Axes		
	Baa	-0.0010	0.075	0.027	0.025	0.0341	0.0030	0.9994
1 O(17)	Bbb	-0.0010	0.069	0.025	0.023	0.4369	0.8994	-0.0176
	Bcc	0.0020	-0.144	-0.051	-0.048	0.8989	-0.4372	-0.0293
	Baa	-0.0049	-0.657	-0.235	-0.219	-0.1047	-0.0787	0.9914
2 C(13)	Bbb	-0.0020	-0.265	-0.094	-0.088	-0.4004	0.9158	0.0304
	Bcc	0.0069	0.922	0.329	0.307	0.9103	0.3938	0.1274
	Baa	-0.0065	-0.867	-0.309	-0.289	-0.4477	0.6965	-0.5608
3 C(13)	Bbb	-0.0014	-0.192	-0.069	-0.064	0.6770	0.6737	0.2963
	Bcc	0.0079	1.060	0.378	0.353	-0.5842	0.2470	0.7731
	Baa	-0.0012	-0.164	-0.059	-0.055	-0.0070	0.2754	0.9613
4 C(13)	Bbb	-0.0010	-0.139	-0.050	-0.046	-0.1478	0.9505	-0.2734
	Bcc	0.0023	0.303	0.108	0.101	0.9890	0.1440	-0.0341
	Baa	-0.0015	-0.208	-0.074	-0.069	0.0273	0.9761	-0.2157
5 C(13)	Bbb	-0.0012	-0.167	-0.060	-0.056	-0.1358	0.2174	0.9666
	Bcc	0.0028	0.375	0.134	0.125	0.9904	0.0029	0.1385
	Baa	-0.0014	-0.186	-0.067	-0.062	-0.2393	-0.2169	0.9464
6 C(13)	Bbb	-0.0009	-0.124	-0.044	-0.041	0.2333	0.9333	0.2729
	Bcc	0.0023	0.311	0.111	0.104	0.9425	-0.2861	0.1727
	Baa	-0.0104	0.749	0.267	0.250	-0.2627	0.5030	0.8234
7 O(17)	Bbb	-0.0048	0.345	0.123	0.115	0.9382	0.3323	0.0964
	Bcc	0.0151	-1.094	-0.390	-0.365	-0.2252	0.7979	-0.5592
	Baa	-0.0033	-1.776	-0.634	-0.592	-0.0892	-0.2697	0.9588
8 H(1)	Bbb	-0.0021	-1.145	-0.408	-0.382	-0.6246	0.7650	0.1571
	Bcc	0.0055	2.920	1.042	0.974	0.7759	0.5848	0.2367

	Baa	-0.0044	-2.351	-0.839	-0.784	0.4501	-0.6008	0.6606
9 H(1)	Bbb	-0.0027	-1.427	-0.509	-0.476	0.0704	0.7614	0.6444
	Bcc	0.0071	3.778	1.348	1.260	0.8902	0.2435	-0.3850
	Baa	-0.0023	0.165	0.059	0.055	0.6019	-0.3744	0.7053
10 O(17)	Bbb	-0.0004	0.030	0.011	0.010	-0.4357	0.5862	0.6830
	Bcc	0.0027	-0.195	-0.070	-0.065	0.6692	0.7185	-0.1897
	Baa	-0.0012	-0.639	-0.228	-0.213	0.7556	-0.4772	0.4487
11 H(1)	Bbb	-0.0010	-0.547	-0.195	-0.182	0.0568	0.7302	0.6809
	Bcc	0.0022	1.186	0.423	0.396	0.6525	0.4890	-0.5789
	Baa	-0.0009	-0.472	-0.168	-0.157	0.1647	-0.6662	0.7274
12 H(1)	Bbb	-0.0008	-0.418	-0.149	-0.139	0.0837	0.7442	0.6627
	Bcc	0.0017	0.889	0.317	0.297	0.9828	0.0482	-0.1783
	Baa	-0.0009	-0.479	-0.171	-0.160	-0.3369	-0.0598	0.9396
13 H(1)	Bbb	-0.0008	-0.436	-0.155	-0.145	-0.0763	0.9964	0.0360
	Bcc	0.0017	0.915	0.327	0.305	0.9384	0.0596	0.3402
	Baa	-0.0008	-0.400	-0.143	-0.134	-0.1464	-0.0612	0.9873
14 H(1)	Bbb	-0.0006	-0.343	-0.122	-0.114	0.2593	0.9608	0.0980
	Bcc	0.0014	0.743	0.265	0.248	0.9546	-0.2704	0.1248
	Baa	-0.0013	-0.690	-0.246	-0.230	-0.3732	0.0978	0.9226
15 H(1)	Bbb	-0.0010	-0.548	-0.195	-0.183	0.3631	0.9305	0.0482
	Bcc	0.0023	1.238	0.442	0.413	0.8538	-0.3529	0.3828
	Baa	-0.0008	0.055	0.020	0.019	0.0265	-0.1342	0.9906
16 O(17)	Bbb	-0.0004	0.032	0.011	0.011	-0.3945	0.9091	0.1337
	Bcc	0.0012	-0.087	-0.031	-0.029	0.9185	0.3944	0.0288
	Baa	-0.1097	-4.233	-1.510	-1.412	0.9461	-0.3217	-0.0363
17 N(14)	Bbb	-0.0963	-3.712	-1.325	-1.238	0.3204	0.9140	0.2490
	Bcc	0.2060	7.945	2.835	2.650	-0.0470	-0.2472	0.9678
	Baa	-0.0220	-2.951	-1.053	-0.984	0.9864	0.1609	0.0331
18 C(13)	Bbb	-0.0030	-0.398	-0.142	-0.133	-0.1602	0.8988	0.4081
	Bcc	0.0250	3.350	1.195	1.117	0.0359	-0.4079	0.9123
	Baa	-0.1435	10.384	3.705	3.464	0.9813	-0.1916	-0.0167
19 O(17)	Bbb	-0.1428	10.333	3.687	3.447	0.1885	0.9413	0.2800
	Bcc	0.2863	-20.717	-7.392	-6.911	-0.0379	-0.2779	0.9598
	Baa	-0.0387	-1.491	-0.532	-0.497	-0.0357	-0.2607	0.9647
20 N(14)	Bbb	0.0160	0.617	0.220	0.206	0.8024	0.5679	0.1832
	Bcc	0.0226	0.874	0.312	0.291	-0.5957	0.7807	0.1890
	Baa	-0.0111	-1.487	-0.531	-0.496	0.6084	0.7620	0.2218
21 C(13)	Bbb	-0.0046	-0.622	-0.222	-0.207	0.7924	-0.5989	-0.1161
	Bcc	0.0157	2.109	0.753	0.704	-0.0444	-0.2464	0.9681
	Baa	-0.0122	0.880	0.314	0.293	0.6556	0.7208	0.2248
22 O(17)	Bbb	-0.0041	0.300	0.107	0.100	0.7528	-0.6472	-0.1202
	Bcc	0.0163	-1.180	-0.421	-0.394	-0.0588	-0.2481	0.9670

	Baa	-0.1190	-15.963	-5.696	-5.325	-0.0374	-0.2461	0.9685
23 C(13)	Bbb	0.0348	4.665	1.665	1.556	0.8715	-0.4823	-0.0890
	Bcc	0.0842	11.298	4.031	3.768	0.4890	0.8407	0.2325
	Baa	-0.2505	-33.618	-11.996	-11.214	-0.6615	0.7347	0.1508
24 C(13)	Bbb	-0.2451	-32.883	-11.734	-10.969	0.7488	0.6355	0.1882
	Bcc	0.4956	66.502	23.730	22.183	-0.0424	-0.2374	0.9705
	Baa	-0.0024	-1.257	-0.449	-0.419	-0.0443	-0.2523	0.9666
25 H(1)	Bbb	0.0009	0.461	0.165	0.154	-0.0314	0.9675	0.2511
	Bcc	0.0015	0.796	0.284	0.266	0.9985	0.0193	0.0508
	Baa	-0.0272	-14.513	-5.178	-4.841	0.6200	0.7533	0.2193
26 H(1)	Bbb	-0.0047	-2.528	-0.902	-0.843	-0.0372	-0.2510	0.9673
	Bcc	0.0319	17.041	6.080	5.684	0.7837	-0.6079	-0.1276
	Baa	-0.6049	-23.330	-8.325	-7.782	-0.4533	0.8681	0.2024
27 N(14)	Bbb	-0.5841	-22.528	-8.039	-7.515	0.8904	0.4301	0.1491
	Bcc	1.1890	45.859	16.364	15.297	-0.0424	-0.2478	0.9679
	Baa	-0.0587	-31.314	-11.174	-10.445	-0.0686	0.9673	0.2443
28 H(1)	Bbb	-0.0109	-5.801	-2.070	-1.935	-0.0418	-0.2475	0.9680
	Bcc	0.0696	37.115	13.243	12.380	0.9968	0.0562	0.0574
	Baa	-0.0007	-0.376	-0.134	-0.125	0.2861	-0.3758	0.8814
29 H(1)	Bbb	-0.0006	-0.324	-0.115	-0.108	-0.3602	0.8102	0.4624
	Bcc	0.0013	0.699	0.249	0.233	0.8879	0.4498	-0.0965
	Baa	-0.0015	-0.811	-0.289	-0.270	-0.0879	-0.2456	0.9654
30 H(1)	Bbb	-0.0013	-0.678	-0.242	-0.226	0.5067	0.8233	0.2556
	Bcc	0.0028	1.489	0.531	0.497	0.8576	-0.5117	-0.0521

4-azidocytidine anion radical (from **3** and **4**) TE = -999.514905194 A.U.

Charge = -1 Multiplicity = 2

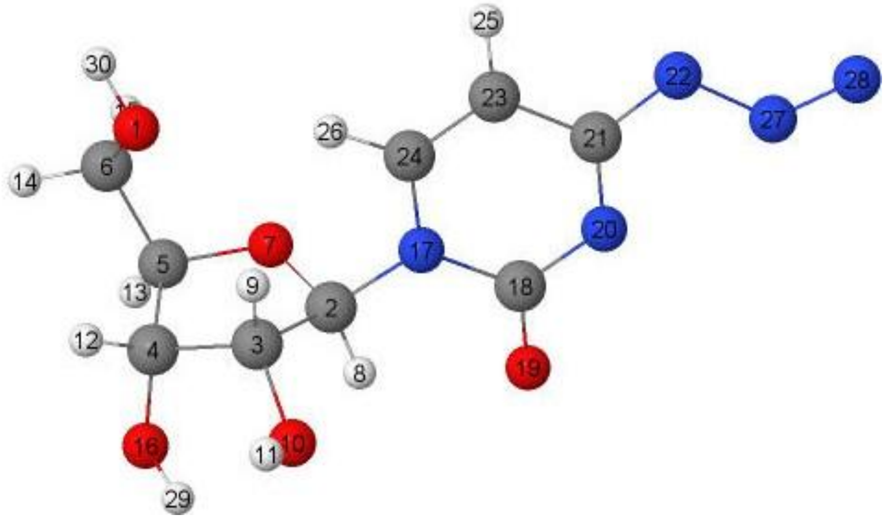

Mulliken charges and spin densities:

		1	2
1	O	-0.559576	0.000008
2	C	0.331000	0.000286
3	C	0.122265	-0.000212
4	C	0.129105	-0.000001
5	C	0.170480	-0.000013
6	C	0.045966	0.000001
7	O	-0.550846	-0.000135
8	H	0.125127	-0.000004
9	H	0.126273	0.000014
10	O	-0.572338	0.000000
11	H	0.345053	0.000001
12	H	0.104738	-0.000011
13	H	0.118387	0.000000
14	H	0.108913	-0.000001
15	H	0.109493	-0.000000
16	O	-0.593927	0.000000
17	N	-0.507300	-0.003233
18	C	0.668264	0.001050
19	O	-0.624561	-0.000396
20	N	-0.626926	0.005690
21	C	0.492440	-0.012071
22	N	-0.510604	0.145352
23	C	-0.192670	0.008569
24	C	0.140989	-0.005121
25	H	0.094607	0.000437
26	H	0.147891	0.000492
27	N	-0.007417	0.177154
28	N	-0.297878	0.682141
29	H	0.324545	0.000000
30	H	0.338504	0.000002

Sum of Mulliken charges = -1.00000 1.00000

Isotropic Fermi Contact Couplings

	Atom	a.u.	MegaHertz	Gauss	10(-4) cm-1
1	O(17)	0.00004	-0.02170	-0.00774	-0.00724
2	C(13)	0.00059	0.66034	0.23562	0.22026
3	C(13)	-0.00008	-0.09417	-0.03360	-0.03141
4	C(13)	0.00002	0.01819	0.00649	0.00607
5	C(13)	0.00002	0.02023	0.00722	0.00675
6	C(13)	-0.00000	-0.00166	-0.00059	-0.00055
7	O(17)	-0.00027	0.16651	0.05942	0.05554
8	H(1)	-0.00001	-0.02674	-0.00954	-0.00892
9	H(1)	0.00000	0.01870	0.00667	0.00624
10	O(17)	0.00001	-0.00675	-0.00241	-0.00225
11	H(1)	-0.00000	-0.00252	-0.00090	-0.00084
12	H(1)	-0.00001	-0.03076	-0.01098	-0.01026
13	H(1)	-0.00000	-0.00179	-0.00064	-0.00060
14	H(1)	0.00000	0.00133	0.00048	0.00045
15	H(1)	-0.00000	-0.00163	-0.00058	-0.00054
16	O(17)	0.00000	-0.00060	-0.00021	-0.00020
17	N(14)	0.00034	0.10960	0.03911	0.03656
18	C(13)	0.00016	0.18292	0.06527	0.06101
19	O(17)	0.00101	-0.61124	-0.21811	-0.20389
20	N(14)	0.00200	0.64711	0.23090	0.21585
21	C(13)	-0.00704	-7.91157	-2.82305	-2.63902
22	N(14)	0.00977	3.15714	1.12655	1.05311
23	C(13)	0.01072	12.05262	4.30067	4.02032
24	C(13)	0.00080	0.90053	0.32133	0.30038
25	H(1)	-0.00006	-0.24631	-0.08789	-0.08216
26	H(1)	0.00021	0.94448	0.33702	0.31505
27	N(14)	0.24310	78.54698	28.02751	26.20045
28	N(14)	0.06792	21.94500	7.83052	7.32006
29	H(1)	0.00000	0.00002	0.00001	0.00001
30	H(1)	0.00000	0.00207	0.00074	0.00069

Anisotropic Spin Dipole Couplings in Principal Axis System

Atom		a.u.	MegaHertz	Gauss	10(-4) cm-1	Axes		
	Baa	-0.0003	0.019	0.007	0.006	-0.1342	-0.6527	0.7456
1 O(17)	Bbb	-0.0003	0.019	0.007	0.006	0.2050	0.7178	0.6653
	Bcc	0.0005	-0.039	-0.014	-0.013	0.9695	-0.2422	-0.0375
	Baa	-0.0007	-0.089	-0.032	-0.030	-0.0994	0.8274	-0.5527
2 C(13)	Bbb	-0.0006	-0.078	-0.028	-0.026	-0.1228	0.5410	0.8320
	Bcc	0.0012	0.167	0.060	0.056	0.9874	0.1506	0.0478
	Baa	-0.0005	-0.061	-0.022	-0.020	-0.1545	0.2148	0.9644
3 C(13)	Bbb	-0.0002	-0.021	-0.008	-0.007	-0.1356	0.9622	-0.2361
	Bcc	0.0006	0.082	0.029	0.027	0.9787	0.1672	0.1195
	Baa	-0.0002	-0.028	-0.010	-0.009	0.1010	-0.5898	0.8012
4 C(13)	Bbb	-0.0002	-0.024	-0.009	-0.008	-0.0381	0.8025	0.5955

		Bcc	0.0004	0.052	0.019	0.017	0.9942	0.0906	-0.0586
		Baa	-0.0002	-0.030	-0.011	-0.010	-0.1602	-0.2164	0.9631
5	C(13)	Bbb	-0.0002	-0.025	-0.009	-0.008	0.0209	0.9747	0.2225
		Bcc	0.0004	0.056	0.020	0.019	0.9869	-0.0558	0.1516
		Baa	-0.0002	-0.026	-0.009	-0.009	0.0982	0.7956	0.5978
6	C(13)	Bbb	-0.0002	-0.025	-0.009	-0.008	-0.1791	-0.5768	0.7970
		Bcc	0.0004	0.051	0.018	0.017	0.9789	-0.1854	0.0859
		Baa	-0.0009	0.066	0.023	0.022	-0.1715	0.7100	-0.6830
7	O(17)	Bbb	0.0000	-0.003	-0.001	-0.001	-0.1586	0.6644	0.7304
		Bcc	0.0009	-0.062	-0.022	-0.021	0.9723	0.2336	-0.0013
		Baa	-0.0004	-0.239	-0.085	-0.080	-0.0442	-0.2593	0.9648
8	H(1)	Bbb	-0.0004	-0.208	-0.074	-0.069	-0.2497	0.9379	0.2407
		Bcc	0.0008	0.446	0.159	0.149	0.9673	0.2302	0.1062
		Baa	-0.0004	-0.192	-0.069	-0.064	0.1611	0.4986	0.8517
9	H(1)	Bbb	-0.0003	-0.172	-0.061	-0.057	-0.0977	0.8668	-0.4890
		Bcc	0.0007	0.364	0.130	0.122	0.9821	0.0044	-0.1884
		Baa	-0.0003	0.022	0.008	0.007	-0.0583	0.7189	0.6927
10	O(17)	Bbb	-0.0003	0.021	0.007	0.007	0.3156	-0.6450	0.6960
		Bcc	0.0006	-0.043	-0.015	-0.014	0.9471	0.2591	-0.1893
		Baa	-0.0003	-0.142	-0.051	-0.047	0.3651	-0.4241	0.8287
11	H(1)	Bbb	-0.0002	-0.131	-0.047	-0.044	-0.0714	0.8748	0.4792
		Bcc	0.0005	0.273	0.097	0.091	0.9282	0.2341	-0.2892
		Baa	-0.0002	-0.086	-0.031	-0.029	0.1028	-0.0233	0.9944
12	H(1)	Bbb	-0.0002	-0.082	-0.029	-0.027	-0.0147	0.9996	0.0249
		Bcc	0.0003	0.168	0.060	0.056	0.9946	0.0172	-0.1024
		Baa	-0.0002	-0.090	-0.032	-0.030	-0.1806	-0.1911	0.9648
13	H(1)	Bbb	-0.0002	-0.086	-0.031	-0.029	-0.0297	0.9816	0.1889
		Bcc	0.0003	0.176	0.063	0.059	0.9831	-0.0055	0.1829
		Baa	-0.0001	-0.073	-0.026	-0.024	-0.1584	-0.5993	0.7847
14	H(1)	Bbb	-0.0001	-0.072	-0.026	-0.024	0.1106	0.7790	0.6173
		Bcc	0.0003	0.144	0.051	0.048	0.9812	-0.1846	0.0572
		Baa	-0.0002	-0.105	-0.037	-0.035	0.3080	0.7831	-0.5402
15	H(1)	Bbb	-0.0002	-0.101	-0.036	-0.034	-0.0046	0.5690	0.8223
		Bcc	0.0004	0.206	0.074	0.069	0.9514	-0.2507	0.1789
		Baa	-0.0002	0.011	0.004	0.004	-0.1636	0.8974	-0.4099
16	O(17)	Bbb	-0.0002	0.011	0.004	0.004	-0.0859	0.4009	0.9121
		Bcc	0.0003	-0.022	-0.008	-0.008	0.9828	0.1844	0.0115
		Baa	-0.0118	-0.453	-0.162	-0.151	-0.0134	-0.3624	0.9319
17	N(14)	Bbb	0.0041	0.160	0.057	0.053	0.1859	0.9149	0.3584
		Bcc	0.0076	0.294	0.105	0.098	0.9825	-0.1780	-0.0551
		Baa	-0.0026	-0.346	-0.124	-0.116	-0.0366	-0.3143	0.9486
18	C(13)	Bbb	-0.0016	-0.215	-0.077	-0.072	-0.3974	0.8755	0.2747
		Bcc	0.0042	0.562	0.200	0.187	0.9169	0.3670	0.1569

	Baa	-0.0044	0.316	0.113	0.105	-0.0073	-0.3809	0.9246
19 O(17)	Bbb	-0.0010	0.074	0.026	0.025	-0.5356	0.7823	0.3180
	Bcc	0.0054	-0.390	-0.139	-0.130	0.8444	0.4929	0.2097
	Baa	-0.0360	-1.387	-0.495	-0.463	-0.0047	-0.3845	0.9231
20 N(14)	Bbb	-0.0029	-0.113	-0.040	-0.038	0.8504	0.4841	0.2059
	Bcc	0.0389	1.501	0.536	0.501	-0.5261	0.7860	0.3247
	Baa	-0.0189	-2.535	-0.905	-0.846	-0.0183	-0.3763	0.9263
21 C(13)	Bbb	0.0002	0.025	0.009	0.008	0.0823	0.9228	0.3765
	Bcc	0.0187	2.511	0.896	0.838	0.9964	-0.0831	-0.0141
	Baa	-0.2349	-9.059	-3.232	-3.022	0.8652	0.4727	0.1673
22 N(14)	Bbb	-0.2332	-8.995	-3.210	-3.001	0.0072	-0.3453	0.9385
	Bcc	0.4681	18.054	6.442	6.022	-0.5014	0.8107	0.3022
	Baa	-0.0068	-0.910	-0.325	-0.304	0.3250	0.8859	0.3310
23 C(13)	Bbb	-0.0040	-0.535	-0.191	-0.178	-0.0180	-0.3441	0.9387
	Bcc	0.0108	1.445	0.516	0.482	0.9456	-0.3110	-0.0959
	Baa	-0.0081	-1.091	-0.389	-0.364	-0.0227	-0.3603	0.9325
24 C(13)	Bbb	0.0012	0.167	0.060	0.056	-0.0924	0.9296	0.3569
	Bcc	0.0069	0.923	0.329	0.308	0.9955	0.0781	0.0545
	Baa	-0.0032	-1.682	-0.600	-0.561	-0.0072	-0.3482	0.9374
25 H(1)	Bbb	-0.0022	-1.181	-0.421	-0.394	0.6344	0.7230	0.2734
	Bcc	0.0054	2.862	1.021	0.955	0.7730	-0.5966	-0.2157
	Baa	-0.0008	-0.447	-0.159	-0.149	-0.0206	-0.3671	0.9299
26 H(1)	Bbb	-0.0005	-0.260	-0.093	-0.087	0.1034	0.9244	0.3672
	Bcc	0.0013	0.707	0.252	0.236	0.9944	-0.1037	-0.0190
	Baa	-0.3210	-12.382	-4.418	-4.130	-0.0107	-0.3487	0.9372
27 N(14)	Bbb	-0.2695	-10.394	-3.709	-3.467	0.8917	-0.4275	-0.1489
	Bcc	0.5905	22.776	8.127	7.597	0.4526	0.8341	0.3155
	Baa	-0.7870	-30.354	-10.831	-10.125	0.9992	-0.0388	0.0015
28 N(14)	Bbb	-0.6727	-25.944	-9.258	-8.654	-0.0148	-0.3451	0.9384
	Bcc	1.4597	56.298	20.088	18.779	0.0359	0.9378	0.3454
	Baa	-0.0002	-0.087	-0.031	-0.029	0.1705	-0.4992	0.8495
29 H(1)	Bbb	-0.0002	-0.086	-0.031	-0.029	-0.1854	0.8305	0.5252
	Bcc	0.0003	0.172	0.062	0.058	0.9678	0.2470	-0.0490
	Baa	-0.0002	-0.109	-0.039	-0.037	-0.0454	-0.2942	0.9547
30 H(1)	Bbb	-0.0002	-0.107	-0.038	-0.036	0.3486	0.8909	0.2911
	Bcc	0.0004	0.217	0.077	0.072	0.9362	-0.3460	-0.0621

4-Azido-1-methylcytosine anion radical (for **3** and **4**)

B3lyp/6-31g** scrf=pcm opt   Charge = -1 Multiplicity = 2

TE = -542.597512574 A.U.

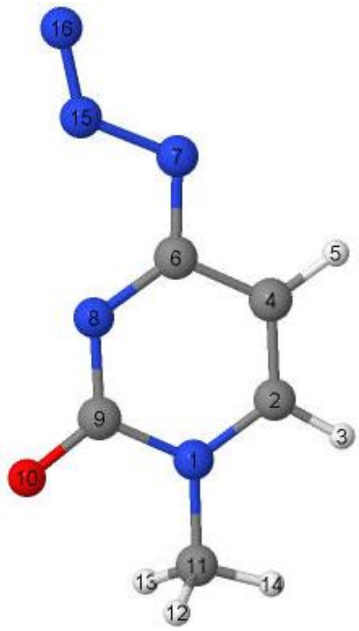

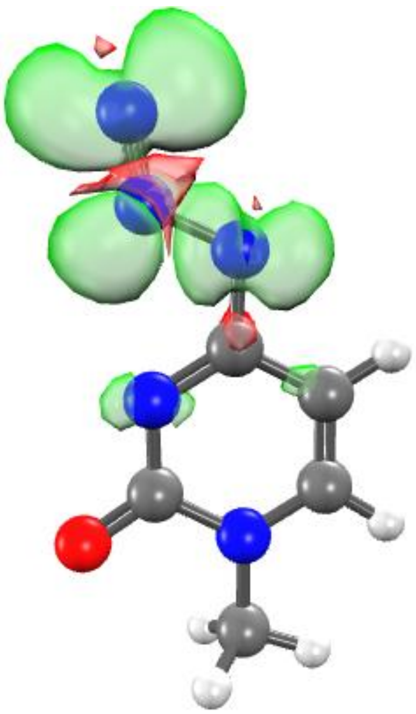

Mulliken charges and spin densities:

		1	2
1	N	-0.472098	-0.003898
2	C	0.143198	-0.004119
3	H	0.135120	0.000610
4	C	-0.188138	0.008526
5	H	0.090852	0.000420
6	C	0.491478	-0.011871
7	N	-0.517963	0.153672
8	N	-0.630245	0.006362
9	C	0.659893	0.001184
10	O	-0.629405	-0.000305
11	C	-0.162901	0.000543
12	H	0.133018	-0.000209
13	H	0.132960	-0.000211
14	H	0.123275	0.000107
15	N	-0.013799	0.168494
16	N	-0.295246	0.680698

Sum of Mulliken charges = -1.00000   1.00000

Isotropic Fermi Contact Couplings

	Atom	a.u.	MegaHertz	Gauss	10(-4) cm-1
1	N(14)	0.00011	0.03449	0.01231	0.01150
2	C(13)	0.00124	1.38921	0.49570	0.46339
3	H(1)	0.00023	1.02377	0.36531	0.34149
4	C(13)	0.01170	13.15417	4.69373	4.38776
5	H(1)	-0.00004	-0.16525	-0.05897	-0.05512
6	C(13)	-0.00743	-8.34918	-2.97920	-2.78499
7	N(14)	0.00969	3.13063	1.11709	1.04426
8	N(14)	0.00198	0.64122	0.22880	0.21389
9	C(13)	0.00027	0.29968	0.10693	0.09996
10	O(17)	0.00099	-0.60228	-0.21491	-0.20090
11	C(13)	0.00065	0.73581	0.26256	0.24544
12	H(1)	-0.00012	-0.51842	-0.18498	-0.17293
13	H(1)	-0.00012	-0.52192	-0.18623	-0.17409
14	H(1)	0.00001	0.05517	0.01968	0.01840
15	N(14)	0.24226	78.27413	27.93016	26.10944
16	N(14)	0.06722	21.72026	7.75033	7.24510

Anisotropic Spin Dipole Couplings in Principal Axis System

Atom		a.u.	MegaHertz	Gauss	10(-4) cm-1	Axes		
	Baa	-0.0135	-0.520	-0.186	-0.173	-0.0000	0.0000	1.0000
1 N(14)	Bbb	0.0049	0.188	0.067	0.063	0.3865	0.9223	-0.0000
	Bcc	0.0086	0.332	0.118	0.111	0.9223	-0.3865	0.0000
	Baa	-0.0073	-0.984	-0.351	-0.328	-0.0001	0.0001	1.0000
2 C(13)	Bbb	0.0007	0.099	0.035	0.033	0.0755	0.9971	-0.0000
	Bcc	0.0066	0.885	0.316	0.295	0.9971	-0.0755	0.0001
	Baa	-0.0009	-0.457	-0.163	-0.153	-0.0001	-0.0002	1.0000
3 H(1)	Bbb	-0.0005	-0.286	-0.102	-0.095	0.3152	0.9490	0.0002

	Bcc	0.0014	0.743	0.265	0.248	0.9490	-0.3152	-0.0000
	Baa	-0.0069	-0.928	-0.331	-0.309	0.5117	0.8592	0.0001
4 C(13)	Bbb	-0.0051	-0.678	-0.242	-0.226	-0.0000	-0.0001	1.0000
	Bcc	0.0120	1.606	0.573	0.536	0.8592	-0.5117	-0.0000
	Baa	-0.0033	-1.757	-0.627	-0.586	0.0000	-0.0001	1.0000
5 H(1)	Bbb	-0.0023	-1.205	-0.430	-0.402	0.7769	0.6296	0.0000
	Bcc	0.0056	2.962	1.057	0.988	-0.6296	0.7769	0.0001
	Baa	-0.0185	-2.477	-0.884	-0.826	0.0001	-0.0000	1.0000
6 C(13)	Bbb	-0.0001	-0.015	-0.005	-0.005	0.2453	0.9695	-0.0000
	Bcc	0.0186	2.492	0.889	0.831	0.9695	-0.2453	-0.0001
	Baa	-0.2441	-9.413	-3.359	-3.140	0.9434	0.3315	-0.0072
7 N(14)	Bbb	-0.2430	-9.373	-3.344	-3.126	0.0068	0.0023	1.0000
	Bcc	0.4871	18.786	6.703	6.266	-0.3315	0.9435	0.0001
	Baa	-0.0366	-1.411	-0.503	-0.471	-0.0000	0.0001	1.0000
8 N(14)	Bbb	-0.0038	-0.146	-0.052	-0.049	0.9406	0.3396	0.0000
	Bcc	0.0404	1.557	0.555	0.519	-0.3396	0.9406	-0.0001
	Baa	-0.0024	-0.319	-0.114	-0.106	-0.0000	-0.0001	1.0000
9 C(13)	Bbb	-0.0018	-0.240	-0.086	-0.080	-0.2201	0.9755	0.0001
	Bcc	0.0042	0.558	0.199	0.186	0.9755	0.2201	0.0000
	Baa	-0.0038	0.275	0.098	0.092	0.0010	-0.0003	1.0000
10 O(17)	Bbb	-0.0011	0.081	0.029	0.027	-0.3556	0.9346	0.0006
	Bcc	0.0049	-0.356	-0.127	-0.119	0.9346	0.3556	-0.0008
	Baa	-0.0006	-0.087	-0.031	-0.029	0.0001	0.0033	1.0000
11 C(13)	Bbb	-0.0005	-0.072	-0.026	-0.024	-0.0198	0.9998	-0.0033
	Bcc	0.0012	0.158	0.057	0.053	0.9998	0.0198	-0.0001
	Baa	-0.0004	-0.214	-0.076	-0.071	-0.1239	0.1634	0.9788
12 H(1)	Bbb	-0.0004	-0.206	-0.074	-0.069	0.0135	0.9865	-0.1629
	Bcc	0.0008	0.420	0.150	0.140	0.9922	0.0070	0.1244

	Baa	-0.0004	-0.214	-0.076	-0.071	0.1241	-0.1688	0.9778
13 H(1)	Bbb	-0.0004	-0.206	-0.074	-0.069	0.0149	0.9856	0.1682
	Bcc	0.0008	0.420	0.150	0.140	0.9922	0.0064	-0.1248
	Baa	-0.0004	-0.201	-0.072	-0.067	-0.0003	0.0008	1.0000
14 H(1)	Bbb	-0.0003	-0.167	-0.060	-0.056	0.1687	0.9857	-0.0007
	Bcc	0.0007	0.369	0.132	0.123	0.9857	-0.1687	0.0005
	Baa	-0.3115	-12.013	-4.287	-4.007	-0.0002	-0.0003	1.0000
15 N(14)	Bbb	-0.2658	-10.249	-3.657	-3.419	0.7823	-0.6228	-0.0000
	Bcc	0.5772	22.263	7.944	7.426	0.6228	0.7823	0.0003
	Baa	-0.7860	-30.314	-10.817	-10.112	0.9755	-0.2202	0.0002
16 N(14)	Bbb	-0.6713	-25.890	-9.238	-8.636	-0.0003	-0.0002	1.0000
	Bcc	1.4573	56.204	20.055	18.748	0.2202	0.9755	0.0003

σ-iminyl radical from 6-azidouridine (**2**)   TE = -965.747764412 A.U.

Charge = 0 Multiplicity = 2

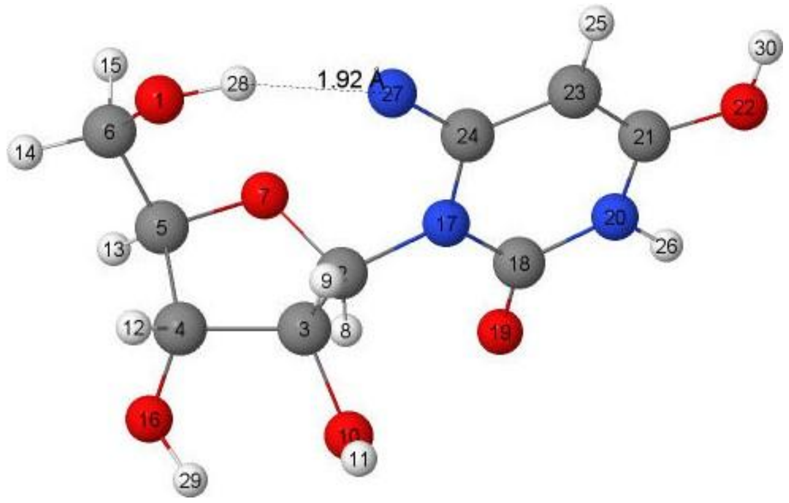

Mulliken charges and spin densities:

		1	2
1	O	-0.605066	0.010479
2	C	0.331139	-0.000142
3	C	0.127649	0.003273
4	C	0.125479	0.000800
5	C	0.162669	-0.000255
6	C	0.043407	0.000252
7	O	-0.543485	0.020682
8	H	0.143248	0.001477
9	H	0.129863	-0.001752
10	O	-0.570953	0.001073
11	H	0.347095	-0.000044
12	H	0.108847	-0.000073
13	H	0.116764	0.000523
14	H	0.115725	0.000412
15	H	0.100479	-0.000029
16	O	-0.590539	0.000092
17	N	-0.593297	0.038077
18	C	0.807226	0.002898
19	O	-0.548828	0.004571
20	N	-0.615806	-0.006056
21	C	0.595986	-0.015713
22	O	-0.535010	-0.000799
23	C	-0.215175	0.059407
24	C	0.582813	-0.106092
25	H	0.138033	-0.001387
26	H	0.321288	0.000158
27	N	-0.524513	0.995323
28	H	0.345482	-0.007355
29	H	0.325667	0.000156
30	H	0.373813	0.000044

Sum of Mulliken charges = -0.00000 1.00000

Isotropic Fermi Contact Couplings

	Atom	a.u.	MegaHertz	Gauss	10(-4) cm-1
1	O(17)	-0.00226	1.36929	0.48860	0.45675
2	C(13)	-0.00172	-1.93789	-0.69149	-0.64641
3	C(13)	0.00149	1.67500	0.59768	0.55872
4	C(13)	0.00023	0.26203	0.09350	0.08740
5	C(13)	-0.00019	-0.21346	-0.07617	-0.07120
6	C(13)	-0.00006	-0.06968	-0.02486	-0.02324
7	O(17)	0.01443	-8.74910	-3.12189	-2.91838
8	H(1)	0.00092	4.12347	1.47136	1.37544
9	H(1)	0.00089	3.97602	1.41874	1.32626
10	O(17)	0.00004	-0.02158	-0.00770	-0.00720
11	H(1)	-0.00002	-0.08555	-0.03053	-0.02854
12	H(1)	-0.00003	-0.13257	-0.04731	-0.04422
13	H(1)	0.00030	1.32273	0.47198	0.44122
14	H(1)	0.00021	0.94102	0.33578	0.31389
15	H(1)	0.00000	0.01708	0.00609	0.00570
16	O(17)	0.00024	-0.14571	-0.05199	-0.04860
17	N(14)	0.06742	21.78356	7.77291	7.26621
18	C(13)	0.00392	4.40554	1.57201	1.46953
19	O(17)	0.00016	-0.09706	-0.03463	-0.03238
20	N(14)	-0.00169	-0.54559	-0.19468	-0.18199
21	C(13)	0.00926	10.40529	3.71287	3.47083
22	O(17)	-0.00099	0.60289	0.21513	0.20110
23	C(13)	0.06357	71.46565	25.50072	23.83838
24	C(13)	-0.03159	-35.51651	-12.67317	-11.84703
25	H(1)	-0.00059	-2.65752	-0.94827	-0.88645
26	H(1)	0.00010	0.45503	0.16237	0.15178
27	N(14)	0.11005	35.55836	12.68811	11.86099
28	H(1)	-0.00097	-4.32606	-1.54364	-1.44302
29	H(1)	0.00005	0.20652	0.07369	0.06889
30	H(1)	0.00011	0.47405	0.16915	0.15813

Anisotropic Spin Dipole Couplings in Principal Axis System

Atom		a.u.	MegaHertz	Gauss	10(-4)cm-1	Axes		
	Baa	-0.0323	2.334	0.833	0.779	0.4973	0.7624	-0.4141
1 O(17)	Bbb	-0.0298	2.156	0.769	0.719	0.2357	0.3406	0.9102
	Bcc	0.0621	-4.491	-1.602	-1.498	0.8350	-0.5502	-0.0104
	Baa	-0.0074	-0.994	-0.355	-0.332	-0.3250	-0.1864	0.9271
2 C(13)	Bbb	-0.0059	-0.798	-0.285	-0.266	0.9074	-0.3375	0.2503
	Bcc	0.0134	1.792	0.639	0.598	0.2663	0.9227	0.2789
	Baa	-0.0084	-1.131	-0.404	-0.377	0.8735	-0.4858	0.0327
3 C(13)	Bbb	-0.0045	-0.602	-0.215	-0.201	-0.0857	-0.0873	0.9925
	Bcc	0.0129	1.733	0.618	0.578	0.4793	0.8697	0.1179
	Baa	-0.0029	-0.390	-0.139	-0.130	-0.4713	0.6383	0.6086
4 C(13)	Bbb	-0.0020	-0.275	-0.098	-0.092	0.3146	-0.5231	0.7921
	Bcc	0.0050	0.664	0.237	0.222	0.8239	0.5648	0.0458

		Baa	-0.0041	-0.544	-0.194	-0.182	-0.3960	0.0666	0.9158
5	C(13)	Bbb	-0.0033	-0.449	-0.160	-0.150	-0.4432	0.8597	-0.2541
		Bcc	0.0074	0.993	0.354	0.331	0.8042	0.5065	0.3109
		Baa	-0.0038	-0.515	-0.184	-0.172	-0.3674	-0.2018	0.9079
6	C(13)	Bbb	-0.0026	-0.350	-0.125	-0.117	0.0279	0.9733	0.2276
		Bcc	0.0064	0.865	0.309	0.289	0.9297	-0.1090	0.3519
		Baa	-0.0643	4.651	1.660	1.551	-0.0417	-0.6002	0.7988
7	O(17)	Bbb	-0.0610	4.417	1.576	1.473	0.9642	-0.2336	-0.1252
		Bcc	0.1253	-9.068	-3.236	-3.025	0.2617	0.7650	0.5885
		Baa	-0.0029	-1.573	-0.561	-0.525	-0.2111	-0.2908	0.9332
8	H(1)	Bbb	-0.0023	-1.213	-0.433	-0.405	0.9652	-0.2126	0.1520
		Bcc	0.0052	2.786	0.994	0.929	0.1542	0.9328	0.3256
		Baa	-0.0109	-5.816	-2.075	-1.940	0.8530	-0.4532	0.2588
9	H(1)	Bbb	-0.0082	-4.368	-1.558	-1.457	0.0171	0.5199	0.8541
		Bcc	0.0191	10.183	3.634	3.397	0.5216	0.7241	-0.4512
		Baa	-0.0053	0.383	0.137	0.128	0.9168	-0.2518	0.3100
10	O(17)	Bbb	-0.0042	0.304	0.108	0.101	-0.2808	0.1457	0.9487
		Bcc	0.0095	-0.687	-0.245	-0.229	0.2840	0.9568	-0.0629
		Baa	-0.0018	-0.981	-0.350	-0.327	0.9490	-0.2942	0.1134
11	H(1)	Bbb	-0.0015	-0.787	-0.281	-0.263	0.0679	0.5418	0.8378
		Bcc	0.0033	1.768	0.631	0.590	0.3079	0.7873	-0.5341
		Baa	-0.0020	-1.062	-0.379	-0.354	0.2531	-0.1099	0.9612
12	H(1)	Bbb	-0.0019	-1.001	-0.357	-0.334	-0.3975	0.8940	0.2069
		Bcc	0.0039	2.063	0.736	0.688	0.8820	0.4344	-0.1825
		Baa	-0.0019	-1.005	-0.359	-0.335	-0.2041	0.8890	-0.4099
13	H(1)	Bbb	-0.0016	-0.864	-0.308	-0.288	-0.5866	0.2241	0.7782
		Bcc	0.0035	1.869	0.667	0.624	0.7837	0.3992	0.4758
		Baa	-0.0017	-0.885	-0.316	-0.295	0.1689	0.7099	-0.6838
14	H(1)	Bbb	-0.0015	-0.799	-0.285	-0.267	-0.1914	0.7041	0.6838
		Bcc	0.0032	1.684	0.601	0.562	0.9669	0.0154	0.2548
		Baa	-0.0027	-1.425	-0.509	-0.475	0.5389	0.6400	-0.5477
15	H(1)	Bbb	-0.0025	-1.331	-0.475	-0.444	-0.2884	0.7511	0.5939
		Bcc	0.0052	2.756	0.983	0.919	0.7915	-0.1622	0.5893
		Baa	-0.0015	0.107	0.038	0.036	-0.6850	0.7131	0.1490
16	O(17)	Bbb	-0.0012	0.089	0.032	0.030	-0.0309	-0.2327	0.9721
		Bcc	0.0027	-0.195	-0.070	-0.065	0.7279	0.6613	0.1814
		Baa	-0.0859	-3.314	-1.183	-1.105	0.0058	-0.3017	0.9534
17	N(14)	Bbb	-0.0519	-2.002	-0.714	-0.668	0.9910	-0.1256	-0.0458
		Bcc	0.1378	5.316	1.897	1.773	0.1336	0.9451	0.2982
		Baa	-0.0081	-1.090	-0.389	-0.364	0.0354	-0.3696	0.9285
18	C(13)	Bbb	-0.0066	-0.880	-0.314	-0.293	0.8003	0.5669	0.1951
		Bcc	0.0147	1.970	0.703	0.657	-0.5985	0.7362	0.3159

		Baa	-0.0196	1.418	0.506	0.473	0.0622	-0.3998	0.9145
19	O(17)	Bbb	-0.0112	0.809	0.289	0.270	0.7429	0.6304	0.2251
		Bcc	0.0308	-2.228	-0.795	-0.743	0.6665	-0.6654	-0.3362
		Baa	-0.0210	-0.808	-0.288	-0.270	-0.0142	-0.3651	0.9309
20	N(14)	Bbb	0.0069	0.268	0.096	0.089	0.6808	0.6783	0.2764
		Bcc	0.0140	0.541	0.193	0.180	0.7323	-0.6376	-0.2389
		Baa	-0.0356	-4.771	-1.702	-1.591	-0.0347	-0.3616	0.9317
21	C(13)	Bbb	0.0057	0.768	0.274	0.256	0.7404	0.6169	0.2670
		Bcc	0.0298	4.003	1.428	1.335	-0.6713	0.6991	0.2463
		Baa	-0.0310	2.246	0.801	0.749	-0.0410	-0.3615	0.9315
22	O(17)	Bbb	0.0009	-0.065	-0.023	-0.022	0.4119	0.8433	0.3454
		Bcc	0.0301	-2.181	-0.778	-0.728	0.9103	-0.3978	-0.1144
		Baa	-0.0381	-5.110	-1.823	-1.704	0.0300	0.9193	0.3923
23	C(13)	Bbb	-0.0269	-3.612	-1.289	-1.205	-0.0116	-0.3921	0.9198
		Bcc	0.0650	8.722	3.112	2.909	0.9995	-0.0322	-0.0011
		Baa	-0.1049	-14.070	-5.021	-4.693	-0.0356	-0.3319	0.9426
24	C(13)	Bbb	-0.0007	-0.094	-0.033	-0.031	0.6777	0.6852	0.2668
		Bcc	0.1056	14.164	5.054	4.725	0.7345	-0.6483	-0.2005
		Baa	-0.0111	-5.938	-2.119	-1.981	-0.0513	-0.3299	0.9426
25	H(1)	Bbb	-0.0063	-3.366	-1.201	-1.123	-0.3391	0.8935	0.2943
		Bcc	0.0174	9.304	3.320	3.104	0.9394	0.3045	0.1577
		Baa	-0.0012	-0.622	-0.222	-0.207	-0.0308	-0.3549	0.9344
26	H(1)	Bbb	-0.0002	-0.114	-0.041	-0.038	0.6281	0.7204	0.2943
		Bcc	0.0014	0.735	0.262	0.245	0.7775	-0.5959	-0.2008
		Baa	-1.0652	-41.082	-14.659	-13.703	0.7017	-0.6828	-0.2036
27	N(14)	Bbb	-0.8287	-31.962	-11.405	-10.661	-0.0652	-0.3461	0.9359
		Bcc	1.8939	73.044	26.064	24.365	0.7095	0.6434	0.2874
		Baa	-0.0214	-11.420	-4.075	-3.809	-0.0734	-0.0834	0.9938
28	H(1)	Bbb	-0.0185	-9.895	-3.531	-3.301	0.3233	0.9407	0.1028
		Bcc	0.0399	21.315	7.606	7.110	0.9435	-0.3288	0.0421
		Baa	-0.0010	-0.526	-0.188	-0.175	-0.5159	0.4560	0.7253
29	H(1)	Bbb	-0.0009	-0.497	-0.177	-0.166	0.6042	-0.4065	0.6853
		Bcc	0.0019	1.023	0.365	0.341	0.6073	0.7917	-0.0658
		Baa	-0.0017	-0.912	-0.325	-0.304	0.0755	0.8535	-0.5155
30	H(1)	Bbb	-0.0017	-0.891	-0.318	-0.297	0.0263	0.5151	0.8567
		Bcc	0.0034	1.804	0.644	0.602	0.9968	-0.0782	0.0164

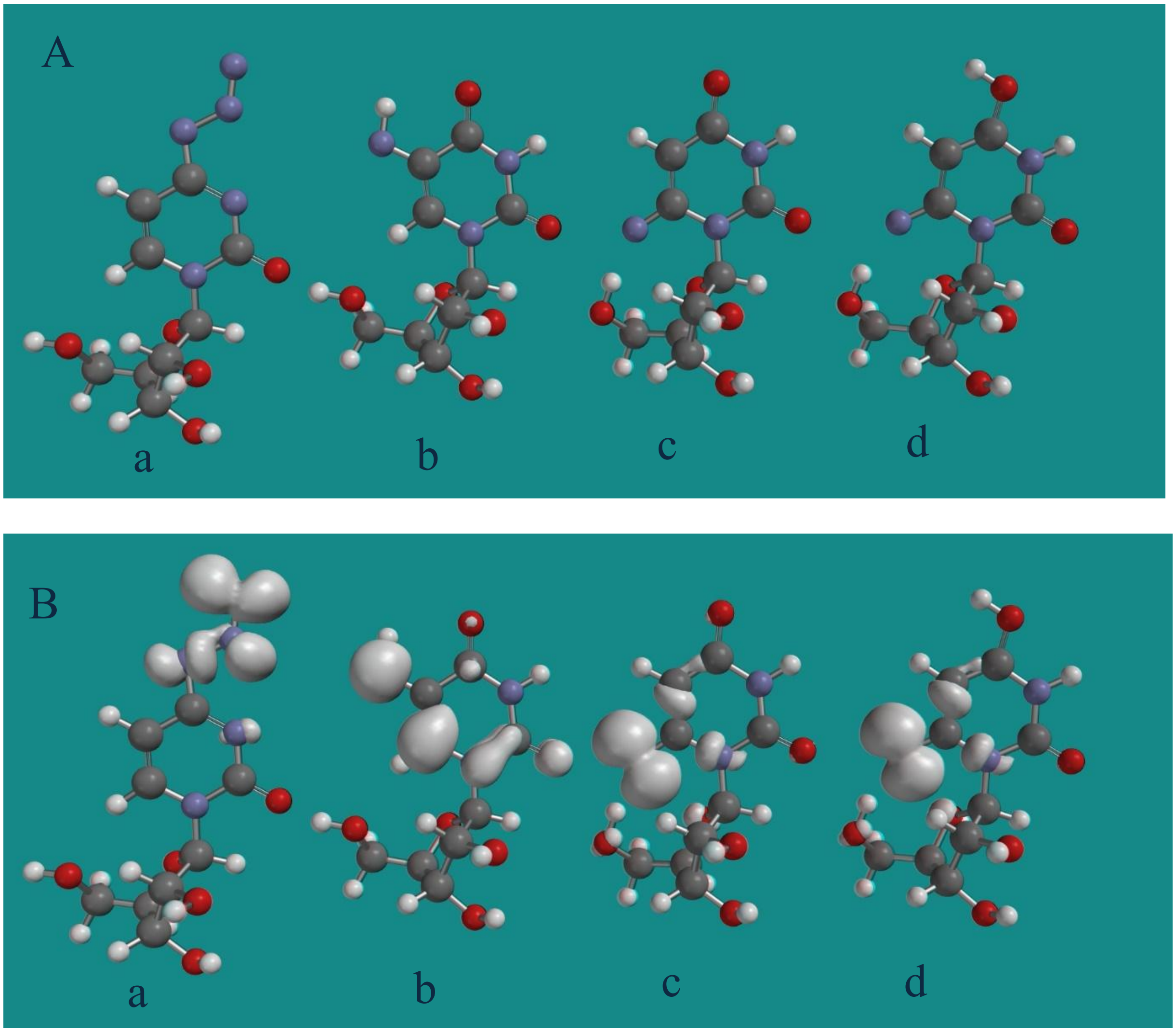

Figure S1. SPARTAN 20 (DFT/B3LYP/6-31+G*, polar solvent)[23] calculated optimized structures and the spin density distributions of radicals obtained from these optimized radical structures formed in the azidopyrimidine ribonucleosides with azido substitution at 4-, 5-, and 6- position of the pyrimidine ring. Figure S1A presents the optimized structures of the radicals and S1B shows the spin densities. Structure a is the 4-azide anion radical ($RN_3\bullet^{-}$) from **3** or **4** (see scheme 3 in the main manuscript), b is the 5-aminyl radical (RNH•), c is the 6-iminyl anion radical with the keto structure, d is the favored σ-type 6-iminyl radical species (R=N•). Protonation at O4 is shown to have little effect on the spin distribution.

4. Ion Chromatography: Methodology

Instrument: The instrument used is the 761 Compact IC system by ΩMetrohm™. The column used is a Shodex SI-90 4E column, with a length of 25 cm and an internal diameter of 4 mm. The detector integrated into this system is a conductivity detector, which measures the electrical conductivity of ionic species in the eluent, providing quantitative analysis of anions.

Type of Chromatography applied: Isocratic chromatography was applied.

Stationary and mobile phase: The stationary phase consists of a polyvinyl alcohol-based anion-exchange resin, which enables selective retention and separation of anions based on their interaction with the fixed positively charged functional groups on the resin.
The mobile phase used is a mixture of sodium carbonate (Na_2CO_3) at a concentration of 3.2 mM and sodium bicarbonate ($NaHCO_3$) at a concentration of 1 mM. This buffered eluent ensures effective separation and elution of anionic species during ion chromatography, maintaining stable pH and ionic strength throughout the run.

Chromatograms:

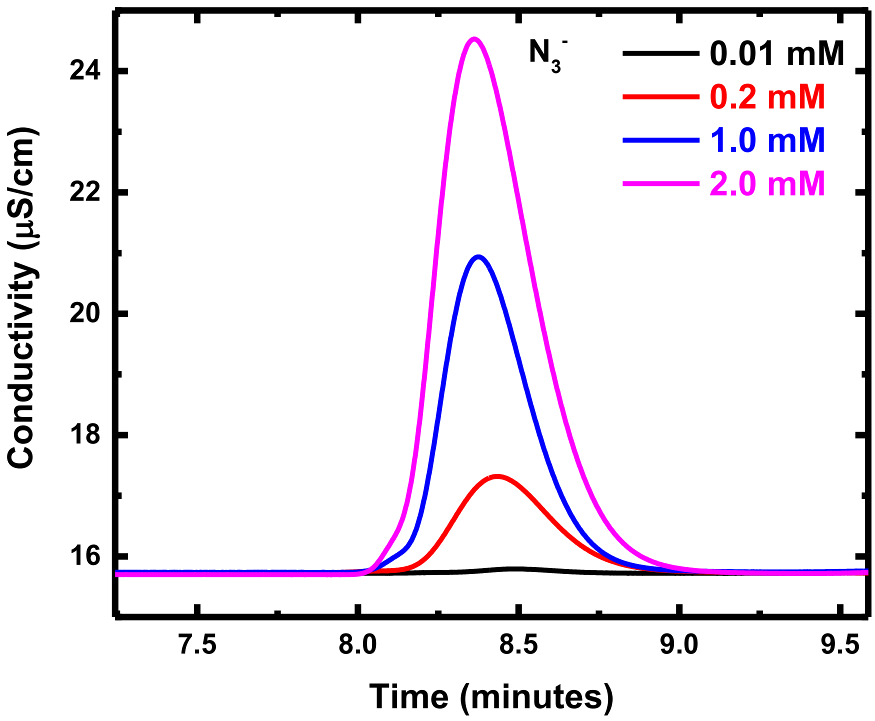

Figure S2. Ion chromatograms of Ar-saturated aqueous solutions of NaN_3 at various concentrations representing the standard intensity vs. concentration curve of N_3^-.

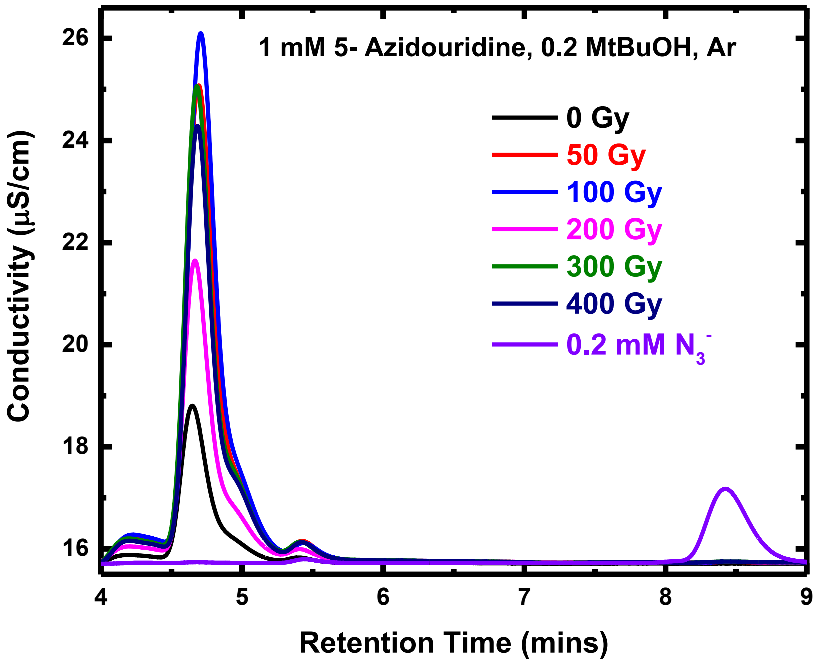

Figure S3. Ion chromatograms of Ar-saturated aqueous solutions of **1** in the presence of 0.2 M *tert*-butanol and gamma-irradiated at various doses. A representative chromatogram of N_3^- is also presented here.

5. References

1. K. J. Divakar and C. B. Reese, 4-(1,2,4-Triazol-1-yl)- and 4-(3-nitro-1,2,4-triazol-1-yl)-1-(β-D-2,3,5-tri-O-acetylarabinofuranosyl)pyrimidin-2(1H)-ones. Valuable intermediates in the synthesis of derivatives of 1-(β-D-arabinofuranosyl)cytosine (ara-C). *J. Chem. Soc., Perkin Trans. 1* **1982**. 1171-1176.
2. M. P. Wallis, N. Mahmood and W. Fraser, Synthesis and anti-HIV activity of C4-modified pyrimidine nucleosides. *Il Falmaco* **1999,** *54*, 83-89.
3. L. P. Kotra, P. Wang, M. G. Bartlett, K. Shanmuganathan, Z. Xu, S. Cavalcanti, M. G. Newton and C. K., Chu, 4-Azido-2-pyrimidinone Nucleosides and Related Chemistry. *J. Org. Chem.* **1997,** *62*, 7267-7271.
4. L. De Napoli, L. Mayol, G. Piccialli, M. Rossi and C. Santacroce, Synthesis of novel pyrimidine nucleoside analogues. *J. Heterocycl. Chem.* **1986,** *23*, 1401-1403.
5. A. Banyasz, K. Tiia-Maaria, A. Muñoz-Losa, S. Rishi, A. Adhikary, M. D. Sevilla, L. Martinez-Fernandez, R. Improta and D. Markovitsi, UV-induced adenine radicals induced in DNA A Tracts: Spectral and Dynamical Characterization. *J. Phys. Chem. Lett.* **2016**, *7*, 3949 − 3953.
6. A. Adhikary, D. Khanduri, V. Pottiboyina, C. T. Rice and M. D. Sevilla, Formation of Aminyl Radicals on Electron Attachment to AZT: Abstraction From the Sugar Phosphate Backbone vs. One-Electron Oxidation of Guanine. *J. Phys. Chem. B* **2010**, *114*, 9289 – 9299.
7. A. Petrovici, A. Adhikary, A. Kumar and M. D. Sevilla, Presolvated Electron Reaction with Methylacetoacetate: Electron Localization, Proton-Deuteron Exchange, and H-atom Abstraction. *Molecules*, **2014**, *19*, 13486 – 13497.
8. A. Adhikary, A. Kumar, D. Khanduri and M. D. Sevilla, The Effect of Base Stacking on The Acid-base Properties of The Adenine Cation Radical [A•$^{+}$] in Solution: ESR and DFT Studies. *J. Am. Chem. Soc.* **2008**, *130*, 10282 – 10292.
9. A. Adhikary, D. Becker, B. J. Palmer, A. N. Heizer and M. D. Sevilla, Direct Formation of The C5′-Radical in The Sugar-Phosphate Backbone of DNA by High Energy Radiation. *J. Phys. Chem. B* **2012**, *116*, 5900 – 5906.
10. S. A. Denisov, S. Ward, V. Shcherbakov, A. D. Stark, R. Kaczmarek, E. Radzikowska-Cieciura, D. Debnath, T. Jacobs, A. Kumar, M. D. Sevilla, P. Pernot, R. Dembinski, M. Mostafavi and A. Adhikary, Modulation of The Directionality of Hole Transfer Between the Base and the Sugar-phosphate Backbone in DNA with the Number of Sulfur Atoms in the Phosphate Group. *J. Phys. Chem. B* **2022**, *126*, 430–442.
11. R. Kaczmarek, S. Ward, D. Debnath, T. Jacobs, A. D. Stark, D. Korczyński, A. Kumar, M. D. Sevilla, S. A. Denisov, V. Shcherbakov, P. Pernot, M. Mostafavi, R. Dembinski and A. Adhikary, One Way Traffic: Base-to-backbone Hole Transfer in Nucleoside Phosphorodithioates. *Chem. Eur. J.* **2020**, *26 (43)*, 9495 –9505. https://chemistry-europe.onlinelibrary.wiley./doi/10.1002/chem.202000247
12. A. Adhikary, A. Kumar, D. Becker and M. D. Sevilla, The guanine Cation Radical: Investigation of Deprotonation States by ESR and DFT. *J. Phys Chem. B* **2006**, *110*, 24170 – 24180.
13. A. Adhikary, A.; D. Khanduri and M. D. Sevilla, Direct Observation of The Hole Protonation State and Hole Localization Site in DNA-Oligomers. *J. Am. Chem. Soc.* **2009**, *131*, 8614 – 8619.
14. A. Adhikary, A. Kumar, S. A. Munafo, D. Khanduri and M. D. Sevilla, Prototropic Equilibria in DNA Containing One-electron Oxidized GC: Intra-duplex vs. Duplex to Solvent Deprotonation. *Phys. Chem. Chem. Phys.* **2010**, *12*, 5353 – 5368.
15. D. Khanduri, A. Adhikary and M. D. Sevilla, Highly Oxidizing Excited States of One-electron Oxidized Guanine in DNA: Wavelength and pH Dependence. *J. Am. Chem. Soc.* **2011**, *133*, 4527 – 4537.
16. A. Adhikary, A. Kumar, R. Rayala, R. M. Hindi, A. Adhikary, S. F. Wnuk and M. D. Sevilla, One-Electron Oxidation of Gemcitabine and Analogs: Mechanism of Formation of C3′ and C2′ Sugar

Radicals. *J. Am. Chem. Soc.* **2014**, *136*, 15646 – 15653.

17. Adhikary, A.; Kumar, A.; Palmer, B. J.; Todd, A. D.; Heizer, A. N.; Sevilla, M. D. Reactions of 5-Methylcytosine Cation Radicals in DNA and Model Systems: Thermal Deprotonation From the 5-Methyl Group vs. Excited State Deprotonation From Sugar. *Int. J. Radiat. Biol.* **2014**, *90*, 433 – 445.
18. F. Wei, C. J. Neal, T. S. Sakthivel, Y. Fu, M. Omer, A. Adhikary, S. Ward, K. M. Ta, S. Moxon, M. Molinari, S. M. Yarmolenko, V. S. Cheong, N. Orlovskaya, R. Ghosh, S. Seal and M. Coathup, A novel approach for the prevention of ionizing radiation-induced bone loss using a designer multifunctional cerium oxide nanozyme. *Bioactive Mater.* **2023**, 21, 547 – 565.
19. A. Adhikary, D. Becker and M. D. Sevilla, Electron Spin Resonance of Radicals in Irradiated DNA. In *Applications of EPR in Radiation Research*, Lund, A.; Shiotani, M., Eds. Springer International Publishing: 2014; pp 299-352.
20. M. J. Frisch, G. W. Trucks, H. B. Schlegel, G. E. Scuseria, M. A. Robb, J. R. Cheeseman, G. Scalmani, V. Barone, B. Mennucci and G. A. Petersson, et al. Gaussian 09; Gaussian, Inc.: Wallingford, CT, 2009.
21. M. J. Raiti and M. D. Sevilla, Density Functional Theory Investigation of The Electronic Structure and Spin Density Distribution in Peroxyl Radicals. *J. Phys. Chem. A* **1999**, *103*, 1619 – 1626.
22. http://jmol.sourceforge.net
23. SPARTAN, version 20; Wavefunction, Inc.: Irvine, CA, 2020.
